# Functional Investigation of a Non-coding Variant Associated with Adolescent Idiopathic Scoliosis in Zebrafish: Elevated Expression of the Ladybird Homeobox Gene Causes Body Axis Deformation

**DOI:** 10.1371/journal.pgen.1005802

**Published:** 2016-01-28

**Authors:** Long Guo, Hiroshi Yamashita, Ikuyo Kou, Aki Takimoto, Makiko Meguro-Horike, Shin-ichi Horike, Tetsushi Sakuma, Shigenori Miura, Taiji Adachi, Takashi Yamamoto, Shiro Ikegawa, Yuji Hiraki, Chisa Shukunami

**Affiliations:** 1 Department of Cellular Differentiation, Institute for Frontier Medical Sciences, Kyoto University, Kyoto, Japan; 2 Department of Molecular Biology and Biochemistry, Division of Basic Life Sciences, Institute of Biomedical and Health Sciences, Hiroshima University, Hiroshima, Japan; 3 Laboratory for Bone and Joint Diseases, RIKEN Center for Integrative Medical Sciences, Tokyo, Japan; 4 Division of Functional Genomics, Advanced Science Research Center, Kanazawa University, Kanazawa, Japan; 5 Department of Mathematical and Life Sciences, Graduate School of Science, Hiroshima University, Higashi-Hiroshima, Hiroshima, Japan; 6 Department of Biomechanics, Research Center for Nano Medical Engineering, Institute for Frontier Medical Sciences, Kyoto University, Kyoto, Japan; The University of Hong Kong, HONG KONG

## Abstract

Previously, we identified an adolescent idiopathic scoliosis susceptibility locus near human *ladybird homeobox 1* (*LBX1*) and *FLJ41350* by a genome-wide association study. Here, we characterized the associated non-coding variant and investigated the function of these genes. A chromosome conformation capture assay revealed that the genome region with the most significantly associated single nucleotide polymorphism (rs11190870) physically interacted with the promoter region of *LBX1-FLJ41350*. The promoter in the direction of *LBX1*, combined with a 590-bp region including rs11190870, had higher transcriptional activity with the risk allele than that with the non-risk allele in HEK 293T cells. The ubiquitous overexpression of human *LBX1* or either of the zebrafish *lbx* genes (*lbx1a*, *lbx1b*, and *lbx2*), but not *FLJ41350*, in zebrafish embryos caused body curvature followed by death prior to vertebral column formation. Such body axis deformation was not observed in transcription activator-like effector nucleases mediated knockout zebrafish of *lbx1b* or *lbx2*. Mosaic expression of *lbx1b* driven by the *GATA2* minimal promoter and the *lbx1b* enhancer in zebrafish significantly alleviated the embryonic lethal phenotype to allow observation of the later onset of the spinal curvature with or without vertebral malformation. Deformation of the embryonic body axis by *lbx1b* overexpression was associated with defects in convergent extension, which is a component of the main axis-elongation machinery in gastrulating embryos. In embryos overexpressing *lbx1b*, *wnt5b*, *a* ligand of the non-canonical Wnt/planar cell polarity (PCP) pathway, was significantly downregulated. Injection of mRNA for *wnt5b* or *RhoA*, a key downstream effector of Wnt/PCP signaling, rescued the defective convergent extension phenotype and attenuated the *lbx1b*-induced curvature of the body axis. Thus, our study presents a novel pathological feature of *LBX1* and its zebrafish homologs in body axis deformation at various stages of embryonic and subsequent growth in zebrafish.

## Author Summary

Scoliosis is the most common type of spinal deformity with a lateral spinal curvature of at least 10 degrees, affecting 2–4% of the population. Scoliosis caused by a primary problem related to the spine itself is classified into congenital scoliosis (CS) and idiopathic scoliosis (IS). Among these, adolescent idiopathic scoliosis (AIS), the most common form of scoliosis, is known as a common polygenic disease. Severe curving of the spine in scoliosis leads to profound psychological and social impacts, but etiology-based therapies have not been established since the precise pathological mechanisms of both IS and CS remain undefined. Previously, we identified an AIS susceptibility locus near human *ladybird homeobox 1* (*LBX1*) by a genome-wide association study. Here, we report the functional characterization of the most significantly associated single nucleotide polymorphism (SNP), rs11190870 and *LBX1* as well as its zebrafish homologues. Overexpression of *LBX1* and zebrafish *lbx* genes caused lateral body curvature in association with the impairment of non-canonical Wnt/planar cell polarity signaling. Thus, our study presents a novel pathological feature of *LBX1* in body axis deformation.

## Introduction

Scoliosis is defined as lateral curvature of the spine with a Cobb angle greater than 10 degrees [1]. It is categorized into congenital, idiopathic, and secondary scoliosis [2]. Congenital scoliosis (CS) is caused by embryonic vertebral malformation that results in deviation of the normal spinal alignment [3]. Idiopathic scoliosis (IS) is a twisting condition of the spine characterized by rotation of the vertebrae without their malformation and is further categorized into infantile, juvenile, and adolescent type by age of onset. Among these forms, adolescent IS (AIS) accounts for 80% of all human scoliosis and develops in 2–4% of children aged between 10 and 16 years across all racial groups [1, 4]. Secondary scoliosis is attributed to a wide variety of causes such as cerebral palsy, paralysis, Duchenne muscular dystrophy, Marfan syndrome, and Ehlers-Danlos syndrome [5–7]. In contrast, the precise disease mechanisms of both IS and CS are understood poorly [8].

Axial skeletal development occurs through a sequential and coordinated series of events regulated by various growth/differentiation factors [2, 9]. During gastrulation, the vertebrate embryo elongates along the anterior-posterior axis through a process called convergent extension [10]. The notochord is then formed ventral to the neural tube as the transient embryonic backbone prior to vertebral bone formation in vertebrates [11]. Following somite segmentation in the paraxial mesoderm, which is formed in a well-defined order along the head to tail axis, the sclerotome derived from the ventral part of the somite eventually gives rise to the vertebrae, the annulus fibrosus of the intervertebral discs, and the rib cage [2]. Any anomalies in these processes are considered to result in the development of both CS and IS.

The role of hereditary or genetic factors especially in the development of AIS has been widely accepted [8]. AIS is a complex polygenic disease influenced by more than one allele at different loci [12]. Indeed, genome-wide association studies identified several novel susceptibility loci including *ladybird homeobox 1* (*LBX1*), *G protein-coupled receptor 126*, zinc finger protein *basonuclin 2*, and *paired box 1* (*PAX1*) [13–16]. Among them, a single nucleotide polymorphism (SNP), rs11190870 in the 3′-flanking region of *LBX1*, has been replicated consistently in independent studies using Chinese [17–19] and Caucasian populations [20].

Human *LBX1* was first identified as a gene with homology to the *ladybird late* (*lbl*) gene in *Drosophila* [21]. The ladybird protein is a member of the homeobox transcription factor family with an engrailed repressor domain [22]. In vertebrates, *Lbx* genes are expressed in the dorsal spinal cord and hindbrain [23], a subpopulation of cardiac neural crest cells [24], muscle precursor cells, and satellite cells of regenerating adult skeletal muscle [25, 26]. Ectopic expression of *LBX1* in chicken somites and limbs activates myogenic markers such as myogenin and *myod*, owing to the expansion of the myoblastic cell population [27]. Previous *in vivo* studies using *Lbx1* knockout mice and *lbx* gene knockdown morphants in zebrafish or *Xenopus* did not reveal phenotypes associated with scoliosis [25, 28–30]. To our knowledge, the pathological features of *Lbx1* and *lbx* genes in body axis deformation have not been explored.

Scoliosis has long been considered to be exclusive to bipedal vertebrates [31]. It has been proposed that the unique human upright posture alters spinal conditions toward the eventual development of scoliosis [32]. Naturally occurring scoliosis is quite rare in quadrupedal vertebrates such as rats and mice [31]. The lack of good animal models *in vivo* has been a major challenge for studying the etiology of scoliosis. Previously, the experimental animal model available for scoliosis research was the young melatonin-deficient chicken, which develops a three-dimensional spinal deformity consisting of lateral curvature after pinealectomy [33]. Recently, it has become clear that several types of fish including zebrafish are suitable for exploring human scoliosis [14, 34–38]. AIS-like scoliosis develops in loss-of-function mutants of *protein tyrosine kinase 7* (*ptk7*) [37] and *kinesin family member 6* in zebrafish [34]. Sharma *et al*. reported that the *PAX1* enhancer locus in humans is associated with susceptibility to IS in females and its enhancer activity is disrupted by IS-associated SNPs [14]. Loss of collagen type VIII alpha 1 function also reportedly causes CS-like vertebral malformations [38].

In this study, we characterized the most significantly associated SNP, rs11190870, using chromosome conformation capture (3C), electrophoretic mobility shift assays (EMSAs), and dual luciferase assays, and then examined the effects of the misregulated expression of *LBX1* on axial skeletal development using zebrafish as an animal model by both gain-of-function and loss-of-function approaches. We demonstrate that the elevated expression of human *LBX1* or zebrafish *lbx1* homologs in zebrafish causes axial developmental defects including defective convergent extension movement and body curvature, which could be attributed to the impairment of non-canonical Wnt/planar cell polarity (PCP) signaling. Some zebrafish transiently overexpressing *lbx1b* survived to develop mild body axis deformation including spinal curvature during larval or juvenile stage. Taken together, our study demonstrated the pathological contribution of *lbx* genes to body axis deformation in zebrafish.

## Results

### Characterization of the AIS-associated region

**Fig 1:**
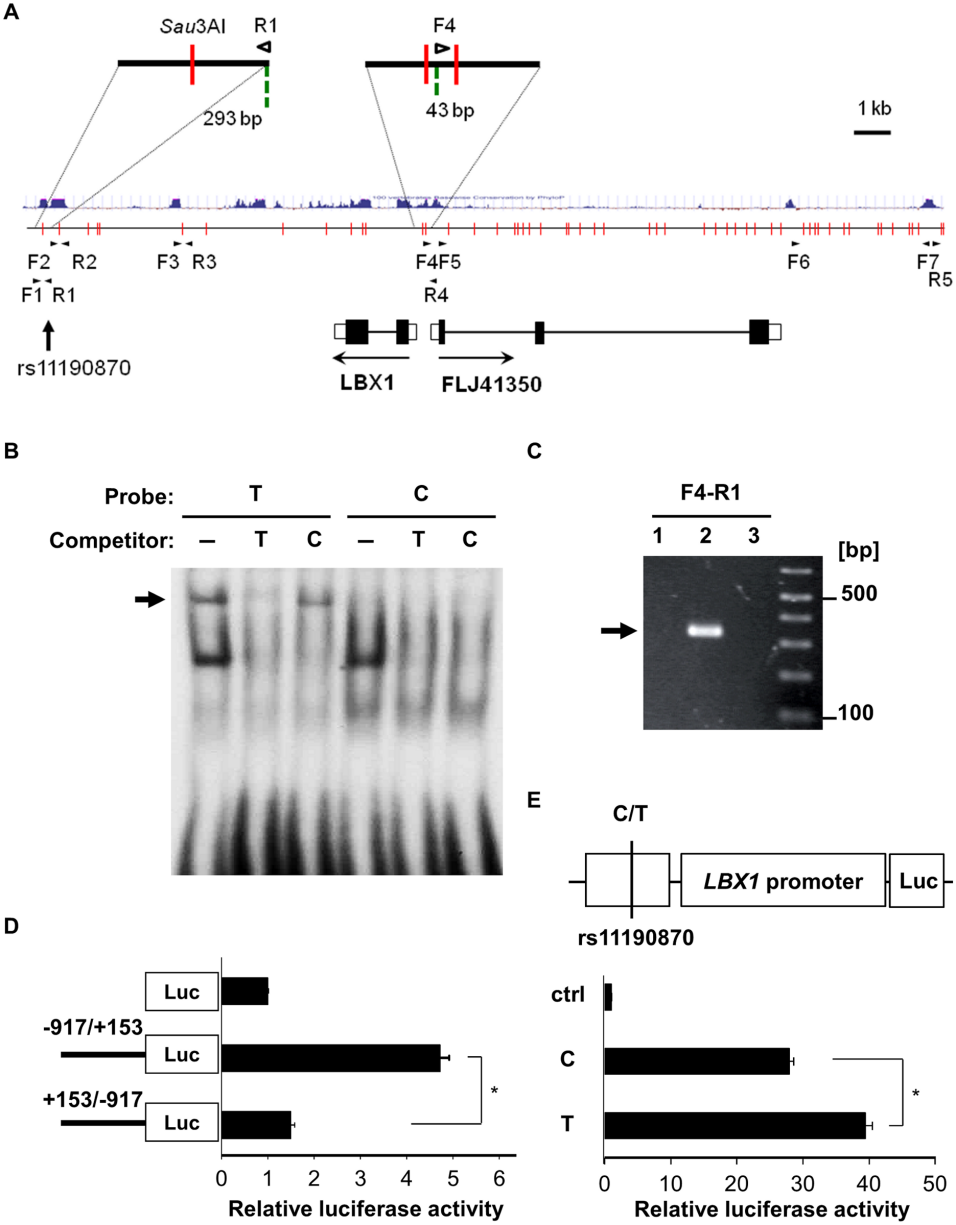
Characterization of rs11190870. (A) A schematic representation of the most significantly associated SNP, rs11190870, and the surrounding genome region with the chromatin fragments obtained by the 3C assay. Cross-linked chromatin was digested with *Sau*3AI (vertical red lines). The green broken lines indicate primer positions. The blue lines indicate vertebrate conservation. The arrowheads indicate PCR primers. (B) Electrophoretic mobility shift assay of rs11190870 using 17-bp DIG-labeled probes for risk (T) and non-risk (C) alleles. Excess unlabeled probes were used as competitors. The arrow indicates that some nuclear factor(s) bound with higher affinity to the risk allele. (C) Physical interaction of rs11190870 and the nearby genomic region with the promoter region of *LBX1-FLJ41350* in the 3C assay (lane 1: rhabdomyosarcoma cells, lane 2: A172 cells, lane 3: HeLa cells). The arrow indicates specific PCR products of the expected sizes derived from the indicated primer sets in (A). (D) Transcriptional activity of the *LBX1-FLJ41350* promoter region. Luciferase activity of the promoter fragment (-917 to +153; direction of *LBX1* transcription) and its reverse fragment (direction of *FLJ41350* transcription) was measured in A172 cells. (E) The effect of the 590-bp conserved region containing rs11190870 on the *LBX1* promoter in HEK 293T cells. Higher activity was observed for the risk allele (T) construct than for the non-risk allele construct (C). Luciferase activity was normalized to the internal control and expressed as a ratio relative to the promoter-less construct (ctrl). The data in (D) and (E) represent the mean ± standard error of two independent experiments. *p < 0.05.

Human *LBX1* and *FLJ41350* are located approximately 0.6 kb apart in a head-to-head arrangement on human chromosome 10, and rs11190870 lies 7.5 kb downstream of *LBX1* (Fig 1A). *FLJ41350* is a hypothetical gene that is found only in the human genome, and its function is uncharacterized [15]. We characterized *FLJ41350* through exon connection and 5′-rapid amplification of cDNA ends. We confirmed that *FLJ41350* is composed of 3 exons, with the predicted translational start site located at exon 1 followed by an open reading frame of 120 amino acids with no known motif (S1 Fig). No orthologs of *FLJ41350* are found in any other species except humans.

To investigate the functional impact of rs11190870, we performed EMSAs and found that some nuclear proteins bound specifically to the genome sequences around rs11190870 with higher affinity to the risk allele than the non-risk allele (Fig 1B). We also analyzed the physical interaction between the genome sequence surrounding rs11190870 and its nearby genome regions using the 3C assay [39] with A172 human glioblastoma cells (A172 cells) (Fig 1C). The 3C assay is a powerful technique for analyzing chromatin organization to reveal the physical interaction between two distal genomic elements [40]. Digestion of cross-linked chromatin with a restriction enzyme and subsequent intra-molecular ligation produces novel junctions between restriction fragments in proximity in the nucleus, which can be detected by PCR. We confirmed that the specific band with primers F4 and R1 was of the expected length (Fig 1C) and corresponded to each primer region by sequencing. This result indicates that the F4 and R1 primer regions are adjacent to each other and that the genome sequence surrounding rs11190870 physically interacts with the promoter region of *LBX1-FLJ41350*.

We then cloned approximately 1 kb of the *LBX1* promoter region (-917 to +153) and evaluated its promoter activity by luciferase assay. In A172 cells, the region had relatively high promoter activity in the direction of *LBX1*, but not in that of *FLJ41350* (Fig 1D). Moreover, the *LBX1* promoter, combined with a 590-bp sequence around rs11190870 that is highly conserved across species, had higher transcriptional activity with the risk allele than with the non-risk allele in HEK 293T cells (Fig 1E). These results suggest that rs11190870 confers AIS susceptibility by upregulating *LBX1* transcription.

### Induction of body curvature by ubiquitous overexpression of *Lbx* genes in zebrafish embryos

**Fig 2:**
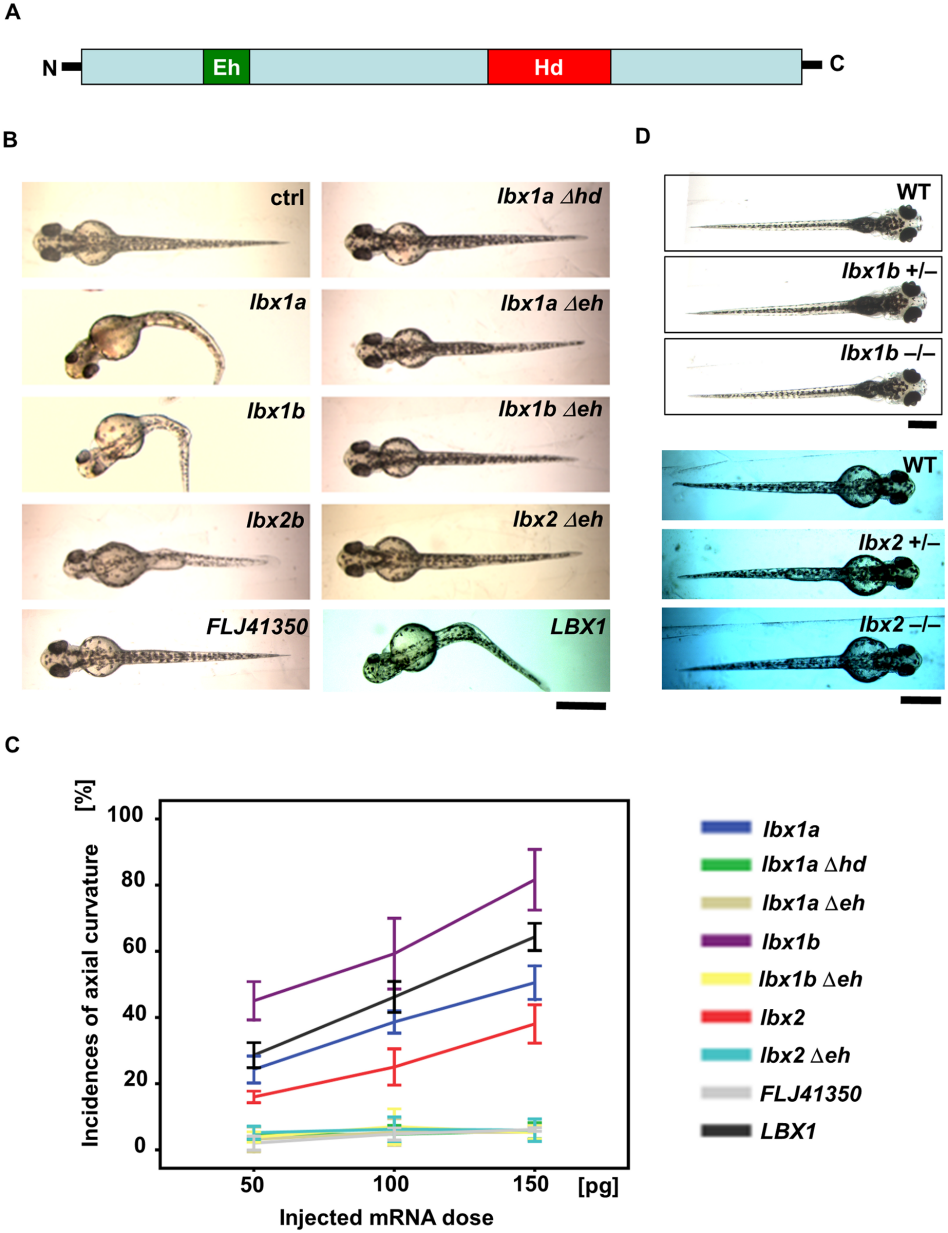
Body curvature induced by injection of *lbx* mRNAs. (A) Protein structure of *Lbx* family members with an engrailed homology domain (Eh) and homeobox domain (Hd). (B) Dorsal views of live embryos (48 hpf) injected with zebrafish *lbx1a*, *lbx1b*, or *lbx2b* mRNA, as well as human *LBX1* or *FLJ41350* mRNA, in comparison with those injected with *lbx1aΔhd* mRNA which lacks the homeodomain, and *lbx1aΔeh*, *lbx1bΔeh*, and *lbx2Δeh* mRNA which lacks the engrailed domain. Buffer was injected as a control (ctrl). Severe curvature was observed in embryos injected with *lbx1a*, *lbx1b*, and *LBX1* mRNA, while mild body curvature was observed in those injected with *lbx2* mRNA, but no curvature was observed in those injected with *lbx* genes lacking the functional domains. Bending of the body axis was not observed in embryos injected with *FLJ41350*. (C) Quantitative analysis of the phenotype of embryos injected with different doses of mRNA. Body curvature occurred in *lbx1b*, *LBX1*, *lbx1a*, *and lbx2* embryos in a dose-dependent manner. *lbx1b* embryos presented with the highest incidence of curvature (45% for 50 pg, 59% for 100 pg, and 82% for 150 pg), and then *LBX1* (29% for 50 pg, 46% for 100 pg, and 64% for 150 pg), *lbx1a* (24% for 50 pg, 39% for 100 pg, and 51% for 150 pg), *and lbx2* (16% for 50 pg, 25% for 100 pg, and 38% for 150 pg). The incidence of curvature was less than 10% in *lbx1a*Δhd, *lbx1a*Δeh, *lbx1b*Δeh, *lbx2*Δeh, and *FLJ41350* embryos regardless of dose. The data represent the mean ± standard error of three independent injections (n = 27–37). (D) Dorsal views of live larva are shown. *lbx1b*^*+/-*^ and *lbx1b*^*-/-*^ mutants as well as *lbx2*^*+/-*^ and *lbx2*^*-/-*^ mutants are comparable to WT with a straight body axis. The scale bar represents 500 μm.

To investigate the effect of the elevated expression of *LBX1* on body axis formation, we performed a series of gain-of-function experiments using zebrafish. We overexpressed zebrafish *lbx1a*, *lbx1b*, *lbx2*, and their mutated genes without the homeodomain or the engrailed homology domain by mRNA injection (Fig 2A and 2B). By 48 hours post-fertilization (hpf), the larvae developed body curvature by the ubiquitous overexpression of any one of these *lbx* genes, but not by that of the mutated genes without the functional domains (Fig 2B). The incidence of body curvature was highest with *lbx1b* overexpression and increased in a dose-dependent manner (Fig 2B and 2C). Notably, some *lbx1b*-overexpressing larvae exhibited notochord deformity and a displaced dorsal melanophore stripe (S2 Fig). In addition, a reduction or complete deletion of the forebrain and eyes was observed in many larvae (S2 Fig). Injection of human *LBX1* mRNA caused body curvature in embryos, but injection of human *FLJ41350* mRNA failed to induce any obvious phenotype related to body axis morphology (Fig 2B).

We also examined the loss-of-function effect on axial development in transcription activator-like effector nuclease (TALEN)-mediated knockout zebrafish (S3 and S4 Figs). Unlike overexpression of *lbx* genes, *lbx1b*^-/-^ and *lbx2*^-/-^ mutant larvae displayed a straight trunk comparable to wild-type larvae (Fig 2D), suggesting the involvement of gain-of-function but not loss-of-function of *lbx1b* in the body curvature phenotype that might be related to scoliosis susceptibility.

**Fig 3:**
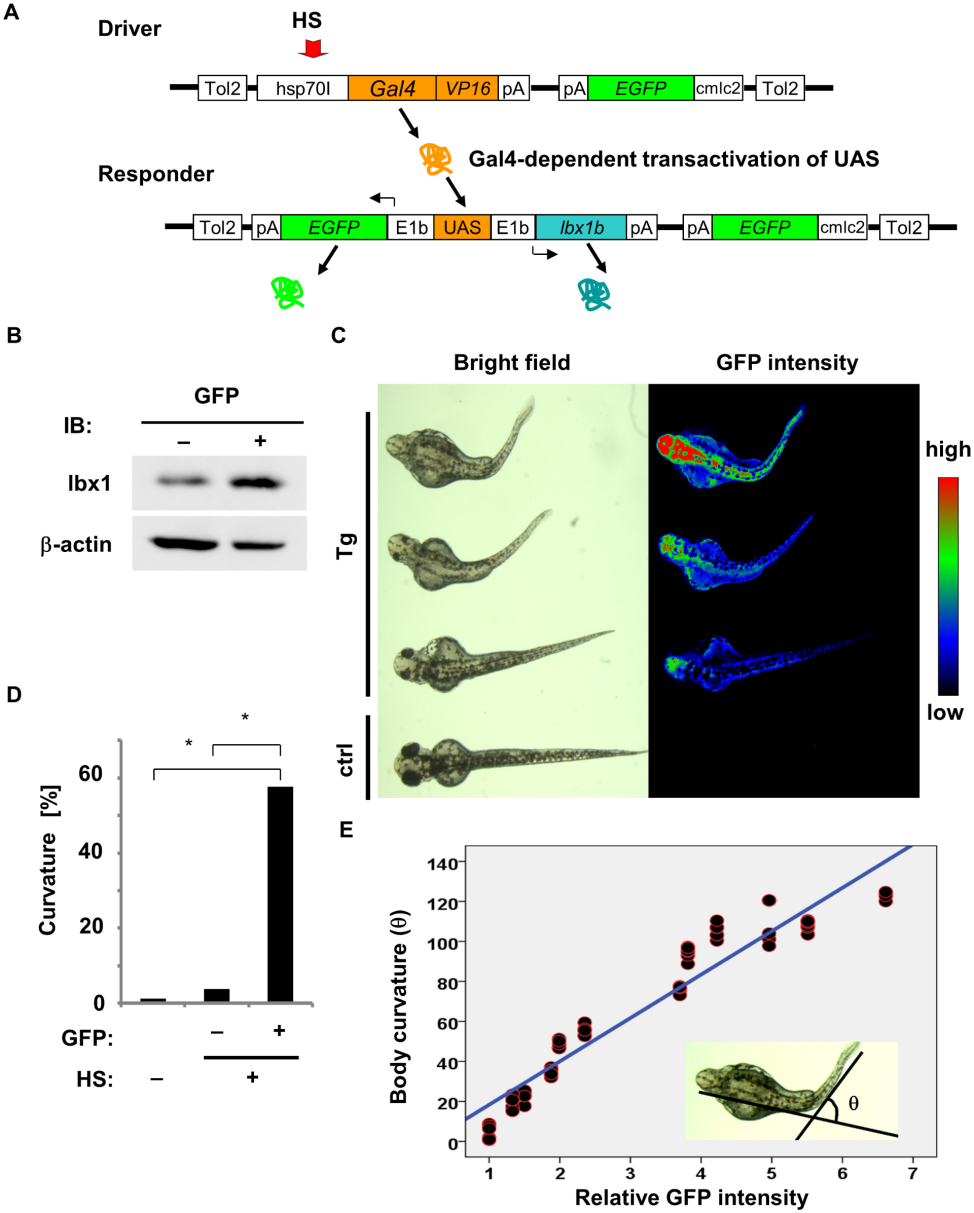
Body curvature induced by *lbx1b* overexpression under the control of the heat shock-inducible *hsp70I* promoter. (A) The constructs in the Gal4/UAS—based bidirectional expression system. The F0 driver transgenic carriers were crossed with the F0 responder transgenic carriers to produce *Tg(hsp*:*Gal-VP*;*EGFP*:*UAS*:*lbx1b*) F1. Heat shock (HS) treatment activated the *hsp70I* promoter in the transgene driver (Driver), which expresses *Gal4-VP16*. Gal4-VP16 protein bound to UAS on the transgene responder (Responder) and activates the expression of *lbx1b* and *EGFP* via E1b minimal promoters. (B) Expression of lbx1 protein in 48 hpf transgenic zebrafish with (GFP^+^) or without HS (GFP^-^). (C) Body curvature of transgenic 48 hpf zebrafish that received HS at 4 hpf. The severity of body curvature was correlated with GFP intensity. (D) Incidence of body curvature at 48 hpf in embryos with (HS+EGFP^+^, n = 85) and without (HS+EGFP^-^, n = 129) EGFP expression and without HS (n = 79). The incidence of curvature was significantly increased in the HS+EGFP^+^ embryos (*p < 0.01). (E) *lbx1b* expression level and body curvature. A linear regression line was obtained between the relative fluorescence intensity of GFP and the severity of body curvature quantified by the angle **θ**: [**θ**/degree] = 21.70 × [relative fluorescence intensity]– 3.37, with the slope coefficient different from 0 (*p* < 0.01). This model accounted for 92.2% of the **θ** variance.

To confirm defective axial development in the established line with uniform expression of *lbx1* in an inducible manner, we employed a Gal4/UAS-based bidirectional expression system for the stable overexpression of *lbx1b* in zebrafish [41]. The F0 driver transgenic carriers were crossed with the F0 responder transgenic carriers to produce *Tg(hsp*:*Gal-VP*;*EGFP*:*UAS*:*lbx1b*) F1 progeny with different copies of the Tol2 insertion. Responding to heat shock, embryos with both driver and responder transgenes expressed *EGFP* and *lbx1b* driven by the E1b promoter (Fig 3A). The positive correlation between the levels of EGFP and *lbx1b* expression was examined in *Tg(hsp*:*Gal-VP*;*EGFP*:*UAS*:*mcherry*) (S5 Fig). Overexpression of *lbx1b* in *Tg(hsp*:*Gal-VP*;*EGFP*:*UAS*:*lbx1b*) after heat shock was confirmed by western blotting (Fig 3B). By 48 hpf, body curvature became evident in *Tg(hsp*:*Gal-VP*;*EGFP*:*UAS*:*lbx1b*) embryos exposed to heat shock at 4 hpf (Fig 3C and 3D). All larvae with *lbx1b* overexpression died within 7 days post-fertilization (dpf). The severity of body curvature was related to the fluorescence intensity of EGFP in a dose-dependent manner (Fig 3C and 3E). Taken together, we conclude that overexpression of *lbx* genes, especially *lbx1b*, induces body curvature in zebrafish embryos.

### Defective convergence and extension movements caused by *lbx1b* overexpression in zebrafish embryos

**Fig 4:**
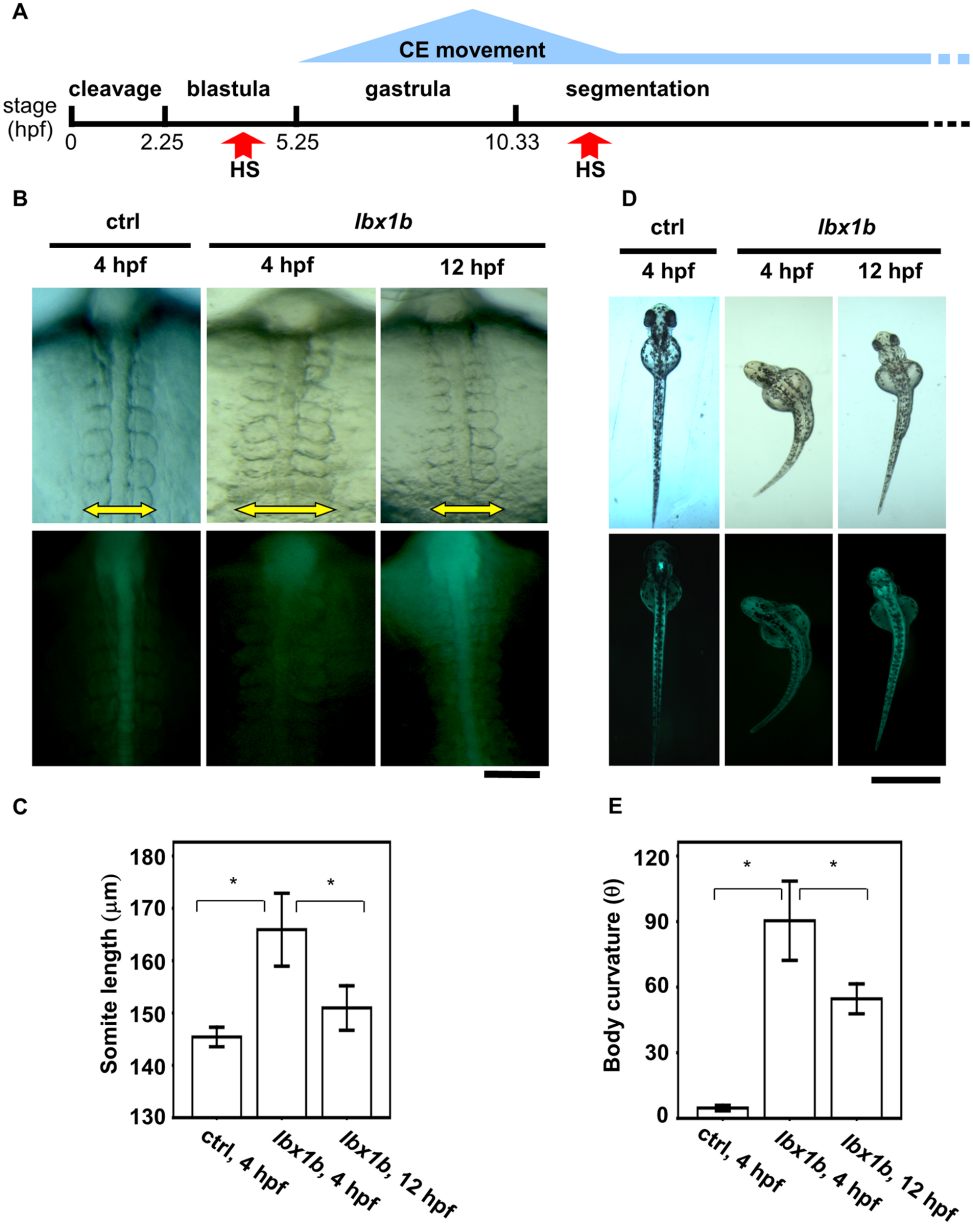
Time-dependent induction of somite mediolateral elongation and body curvature by *lbx1b* overexpression. (A) A schematic diagram of zebrafish early developmental stages. Convergent extension (CE) is mainly involved in gastrulation. (B) Comparison of somite mediolateral length (16 hpf) between *Tg(hsp*:*Gal-VP; UAS*:*EGFP)* with heat shock (HS) treatment at 4 hpf (ctrl 4 hpf), *Tg(hsp*:*Gal-VP; EGFP*:*UAS*:*lbx1b)* with HS at 4 hpf (*lbx1b* 4 hpf), and *Tg(hsp*:*Gal4-VP; EGFP*:*UAS*:*lbx1b)* with HS at 12 hpf (*lbx1b* 12 hpf). A significant elongation of somite mediolateral length (yellow arrow in the upper panels) was observed in the *lbx1b* embryos at 4 hpf. The scale bar represents 100 μm. (C) Quantitative analysis of somite mediolateral length in embryos at 16 hpf. A significant difference was observed between ctrl at 4 hpf (n = 16) and *lbx1b* at 4 hpf (n = 13), and *lbx1b* at 4 hpf (n = 13) and *lbx1b* at 12 hpf (n = 14), *p < 0.01. (D) Comparison of body curvature (48 hpf) between *Tg(hsp*:*Gal-VP; UAS*:*EGFP)* with HS treatment at 4 hpf (ctrl 4 hpf), *Tg(hsp*:*Gal-VP*:*EGFP*:*UAS*:*lbx1b)* with HS at 4 hpf (*lbx1b* 4 hpf), and *Tg(hsp*:*Gal-VP*:*EGFP*:*UAS*:*lbx1b)* with HS at 12 hpf (*lbx1b* 12 hpf). More severe curvature of the body axis was induced in *lbx1b* embryos at 4 hpf than in *lbx1b* embryos at 12 hpf. The scale bar represents 1 mm (E) Quantitative analysis of body curvature at 48 hpf. A significant difference was observed between ctrl 4 hpf (n = 13) and *lbx1b* 4 hpf (n = 10), and between *lbx1b* 4 hpf (n = 10) and *lbx1b* 12 hpf (n = 11), *p < 0.05. The severity of body curvature was quantified by the angle described in the legend of Fig 3. Both driver transgenic and responder transgenic fish were F2 lines.

**Fig 5:**
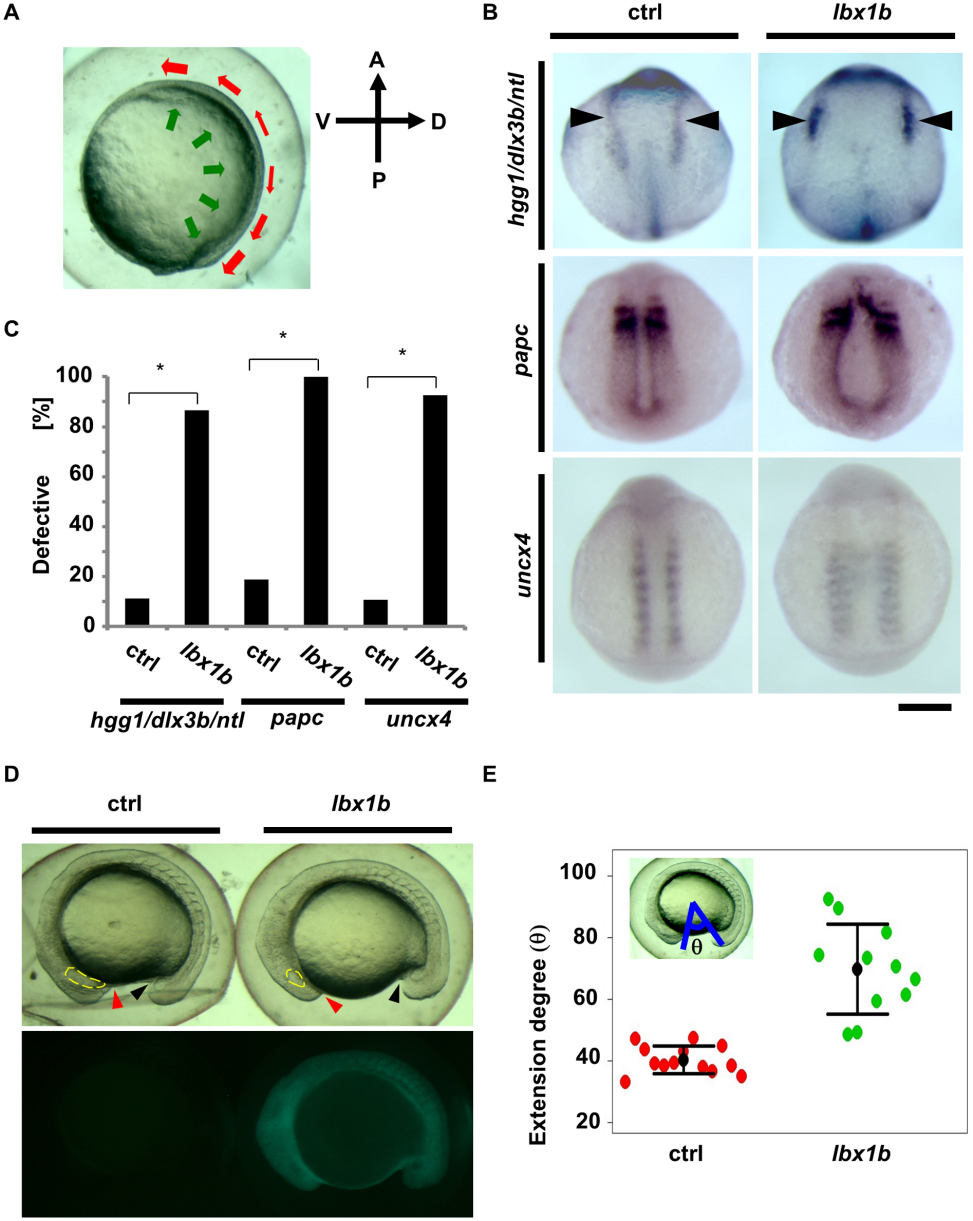
Defective convergent extension in *lbx1b* overexpressing zebrafish embryos. (A) A diagram of convergent extension during zebrafish gastrulation. A, P, D, and V indicate anterior, posterior, dorsal, and ventral sides, respectively. The green and red arrows indicate the directions of convergence and extension movements, respectively. (B) *In situ* hybridization for germ layer markers at the early stages of development. Dorsal views of embryos injected with buffer as control (ctrl) or *lbx1b* mRNA (*lbx1b*). Upper panels show marker expression of *hgg1* (rostral mesoderm), *ntl* (notochord), and *dlx3b* (border between neural and non-neural ectoderm, black arrowheads) at the tail bud stage. Middle panels show the expression of *papc* (paraxial mesoderm) at 11 hpf. Lower panels show the expression of *uncx4* (somite) at 13 hpf. The scale bar represents 200 μm. (C) Incidence of defective expression patterns. Significant differences (*p < 0.01) were observed for the germ layer markers. The numbers of control and *lbx1b* zebrafish embryos were 36 and 30 for *hgg1*/*ntl*/*dlx3b*, 32 and 28 for *papc*, and 28 and 27 for *uncx4*, respectively. (D) Lateral views of 16 hpf normal sibling (ctrl) and *Tg(hsp*:*Gal4-VP16*:*EGFP*:*UAS*:*lbx1b)* (*lbx1b*) embryos upon heat shock at 4 hpf. The red and black arrowheads indicate the border between the yolk and the rostral part and the yolk and the caudal part, respectively. The yellow lines indicate the developing eyes. (E) Quantitative analysis of extension movement. Progression of the movement was quantified by the angle **θ** at 16 hpf for sibling control (ctrl; n = 13) and transgenic (*lbx1b*; n = 11) embryos. Convergent extension movement was significantly delayed in *lbx1b* embryos (*p* < 0.01).

To elucidate the mechanism by which *lbx1* overexpression causes embryonic body curvature, we traced back to the pregastrulation stages. Convergent extension movement during gastrulation (5.25–10.33 hpf) shapes the body axis, narrowing all germ layers in the mediolateral direction and extending them along the anterioposterior axis (Figs 4A and 5A). Embryos overexpressing *lbx1b* in the gastrulation stage showed mediolateral elongation of somites (Fig 4B and 4C), suggesting some perturbations occur in the formation of the body axis due to abnormal convergent extension. Embryos exposed to heat shock at 4 hpf exhibited a more profound convergent extension defect and more severe body curvature than those at 12 hpf (Fig 4B–4E), demonstrating a positive correlation between the extent of defective convergent extension with the severity of body curvature and the presence of a critical time window for *lbx1b* overexpression. *In situ* hybridization for the characteristic markers for the ectoderm or mesoderm revealed a marked delay of convergent movement in embryos overexpressing *lbx1b* (Fig 5B and 5C). Compared with sibling controls, *lbx1b*-overexpressing embryos showed a wider neural ectoderm border (*dlx3b*), broader paraxial mesoderm (*papc*), and mediolateral elongation (*uncx4*) of somites (Fig 5B). We also found a significant delay of extension movement (Fig 5D and 5E), which elongated the embryo from head to tail. By contrast, the expression pattern of a dorsal marker, *chordin* (*chd*), and a ventral marker, *ventral homeobox* (*vox*), in early gastrula was not significantly altered in *lbx1b*-overexpressing embryos (S6 Fig), indicating that dorsoventral patterning was not affected in these embryos. These results indicate that defective convergent extension resulting from elevated *lbx1b* expression during gastrulation provokes impaired body axis formation.

### Downregulation of *wnt5b* in *lbx1b*-overexpressing embryos

**Fig 6:**
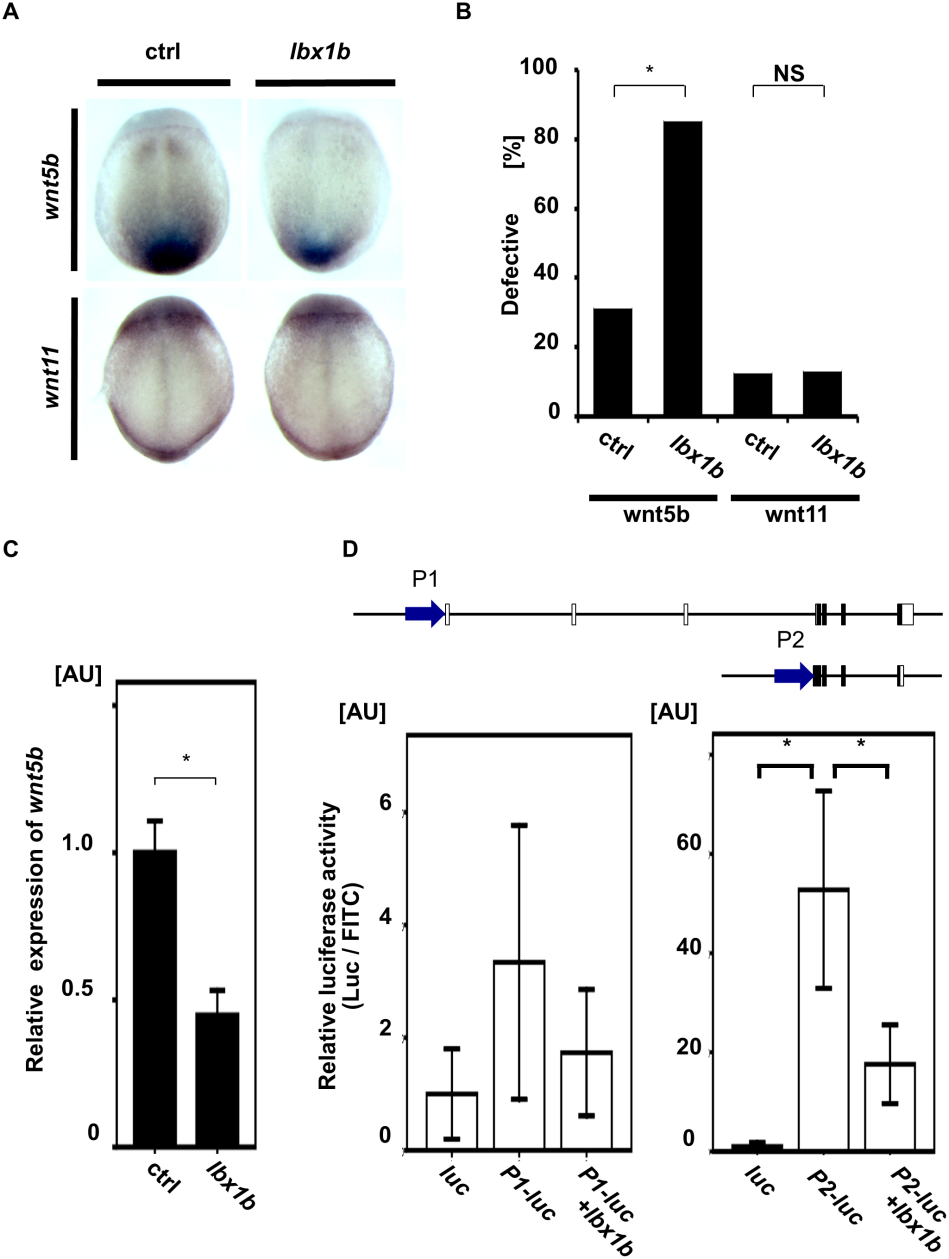
Downregulation of *wnt5b* during gastrulation by *lbx1b* overexpression. (A) *In situ* hybridization for non-canonical Wnt/PCP ligands (*wnt5b* and *wnt11*) in control and *lbx1b*-overexpressing embryos at 90% epiboly. Dorsal views (anterior to the top) for *wnt5b* and *wnt11* of embryos injected with buffer (ctrl) or *lbx1b* mRNA (*lbx1b*). (B) Incidence of the defective expression patterns observed in the embryos shown in panel A. Significant differences (*p < 0.01) were observed for *wnt5b*. The numbers of control and *lbx1b* zebrafish embryos were 22 and 21 for *wnt5b* and 23 and 22 for *wnt11*, respectively. NS: not significant. (C) Decreased *wnt5b* expression in 90% epiboly embryos injected with *lbx1b* mRNA by quantitative RT-PCR assays. *p < 0.01. (D) *In vivo* luciferase assay in 90% epiboly embryos injected with control vector (*luc*) or the putative promoter regions (P1 and P2) of zebrafish *wnt5b*. P2 showed higher transcriptional activity than P1. Co-injection of *lbx1b* mRNA in P2 significantly repressed the transcriptional activity. *p < 0.01. AU, arbitrary unit.

In vertebrates, non-canonical Wnt/PCP signals are mainly involved in the regulation of convergent extension [10]. Loss of function of *wnt5b* or *wnt11*, the ligand for the non-canonical Wnt/PCP signaling pathway, leads to severely defective convergent extension movement in zebrafish [42, 43]. Our *in situ* hybridization study revealed that *wnt5b* was downregulated in gastrulation embryos upon *lbx1b* overexpression (Fig 6A and 6B and S7 Fig). In contrast, no significant change was observed in *wnt11* expression (Fig 6A and 6B and S7 Fig). We also confirmed by quantitative RT-PCR that *wnt5b* expression was significantly downregulated at the gastrulation stage in *lbx1b*-overexpressing embryos (Fig 6C).

We then performed an *in vivo* luciferase assay in zebrafish embryos to analyze the *in vivo* effects of *lbx1b* overexpression on the transcriptional activity of two potential promoter regions of *wnt5b*. Two transcripts encoding 363 (MN1309037) or 380 amino acids were found on the Ensembl website. We tested the sequences upstream of *wnt5b* including these promoters (P1 and P2). In 90% epiboly embryos, the P2 promoter had much stronger transcriptional activity (about 50-fold induction) than the P1 promoter. Co-injection of *lbx1b* mRNA repressed the transcriptional activity of P2 by 66.7% (Fig 6D). Thus, *lbx1b* overexpression during gastrulation downregulated the expression of *wnt5b* largely through repression of the P2 promoter. These results suggest that defective convergent extension caused by the overexpression of *lbx1b* in embryos could be attributed to impairment of non-canonical Wnt/PCP signaling.

### Impairment of non-canonical Wnt/PCP signaling during gastrulation in embryos overexpressing *lbx1b*

**Fig 7:**
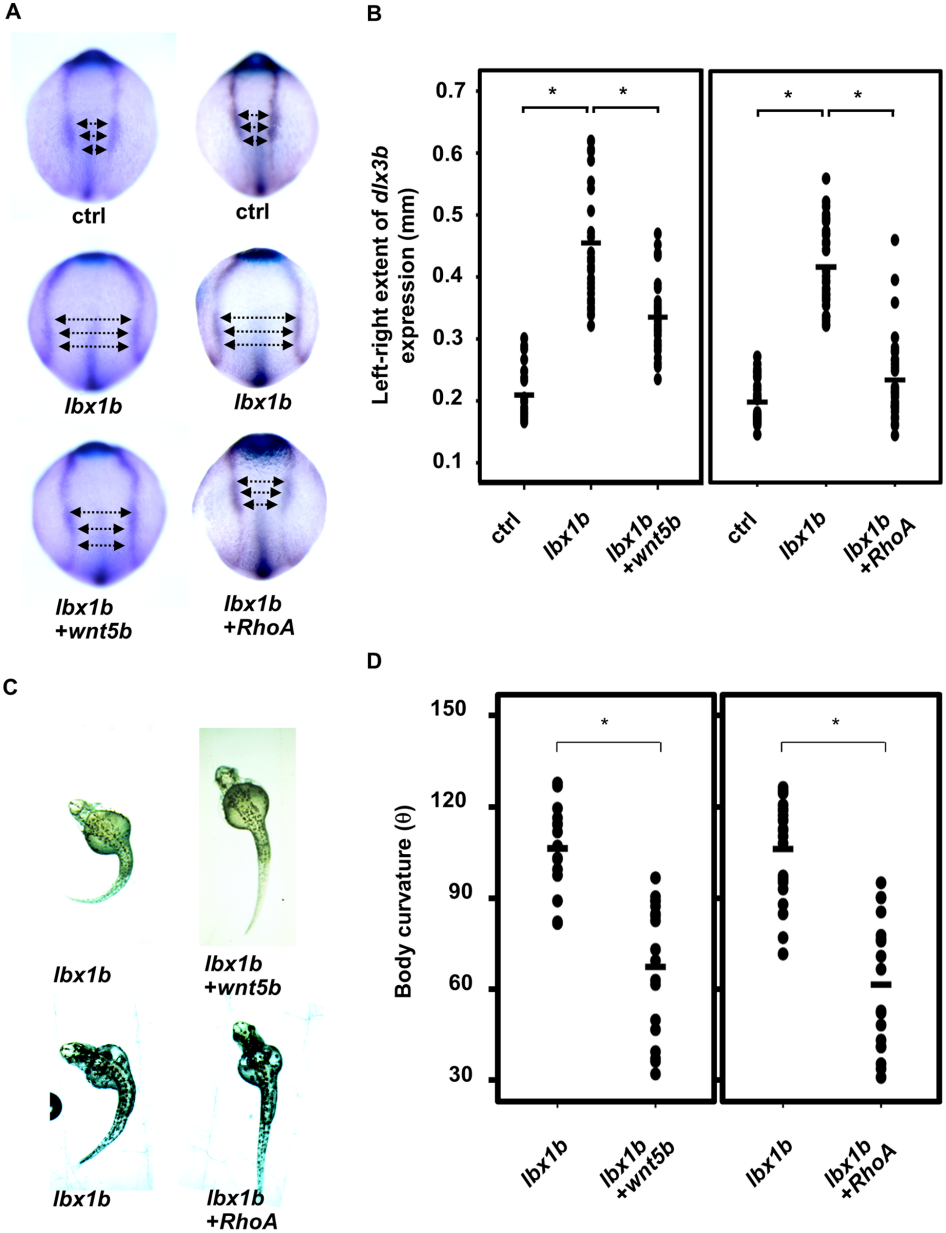
Rescue of defective convergent extension in *lbx1b*-overexpressing embryos by *wnt5b* and *RhoA* mRNA injection. (A) Dorsal view of *dlx3b/hgg1/ntl* expression in tail bud embryos injected with buffer (ctrl), *lbx1b mRNA* (*lbx1b*), *lbx1b* plus zebrafish *wnt5b* mRNA (*lbx1b*+*wnt5b*), or *lbx1b* plus human *RhoA* mRNA (*lbx1b*+*RhoA*). (B) Quantitative analysis of convergent extension movement in the tail bud embryos in (A). (ctrl, n = 26 and 29; *lbx1b*, n = 27 and 33; *lbx1b*+*wnt5b*, n = 27; *lbx1b*+*RhoA*, n = 32). The extent of defective convergent extension was evaluated by measuring the distance between the inner edges of bilateral *dlx3b* expression at 3 regions as indicated by the arrows in panel A. *Wnt5b* or *RhoA* mRNA injection significantly rescued defective convergent extension (*p < 0.01). (C) Dorsal view of 48 hpf *Tg(hsp*:*Gal-VP;EGFP*:*UAS*:*lbx1b)* embryos upon heat shock at 4 hpf with buffer (*lbx1b*), *wnt5b* mRNA (*lbx1b*+*wnt5b*), or *RhoA* mRNA (*lbx1b*+*RhoA*) injection. (D) Quantitative analysis of body curvature in *Tg(hsp*:*Gal4-VP;EGFP*:*UAS*:*lbx1b)* embryos upon heat shock at 4 hpf with buffer injection (*lbx1b*, n = 16 and 21; *lbx1b*+*wnt5b*, n = 18; *lbx1b*+*RhoA*, n = 18).The severity of body curvature was significantly alleviated in *Wnt5b* or *RhoA* mRNA-injected embryos with heat shock at 4 hpf, *p < 0.01. Severity of body curvature was quantified by the angle as described in Fig 3. F2 lines of driver transgenic and responder transgenic fish were used. Injections were performed at doses of 50 pg/embryo for *lbx1b* mRNA, 40 pg/embryo for *wnt5b* mRNA, and 15 pg/embryo for *RhoA* mRNA.

To evaluate further the effect of misregulation of non-canonical Wnt/PCP signaling in defective convergent extension caused by *lbx1b* overexpression, we performed a rescue experiment by overexpressing *wnt5b*, a ligand of the Wnt/PCP pathway. We optimized the amount of *wnt5b* mRNA injection to avoid defects caused by its overexpression in embryos. Defective migration of *dlx3b*-positive cells in embryos injected with *lbx1b* mRNA was rescued by co-injection of *lbx1b* and *wnt5b* mRNA (Fig 7A and 7B). *Wnt5b* mRNA injection mostly rescued the body curvature phenotype in *Tg(hsp*:*Gal-VP;EGFP*:*UAS*:*lbx1b)* with heat shock at 4hpf (Fig 7C and 7D). We further examined whether defects caused by *lbx1b* overexpression can be rescued by overexpressing *RhoA* or *Rac1* small GTPases, both of which are downstream effectors of the Wnt/PCP pathway. *RhoA* rescued both the defective convergent extension and body curvature phenotype (Fig 7A and 7B), whereas *Rac1* failed to rescue the convergent extension defects and body curvature (S8 Fig). Interestingly, *RhoA* overexpression was not effective in larvae with heat shock at 12 hpf (S9 Fig). These results demonstrate that impairment of non-canonical Wnt/PCP signaling, especially the *wnt5b*/*RhoA* pathway, caused by *lbx1b* overexpression, contributes to defective convergent extension and curvature of the body axis.

### Scoliosis in zebrafish expressing *lbx1b* under the control of the *GATA2* minimal promoter and the *lbx1b* enhancer

**Fig 8:**
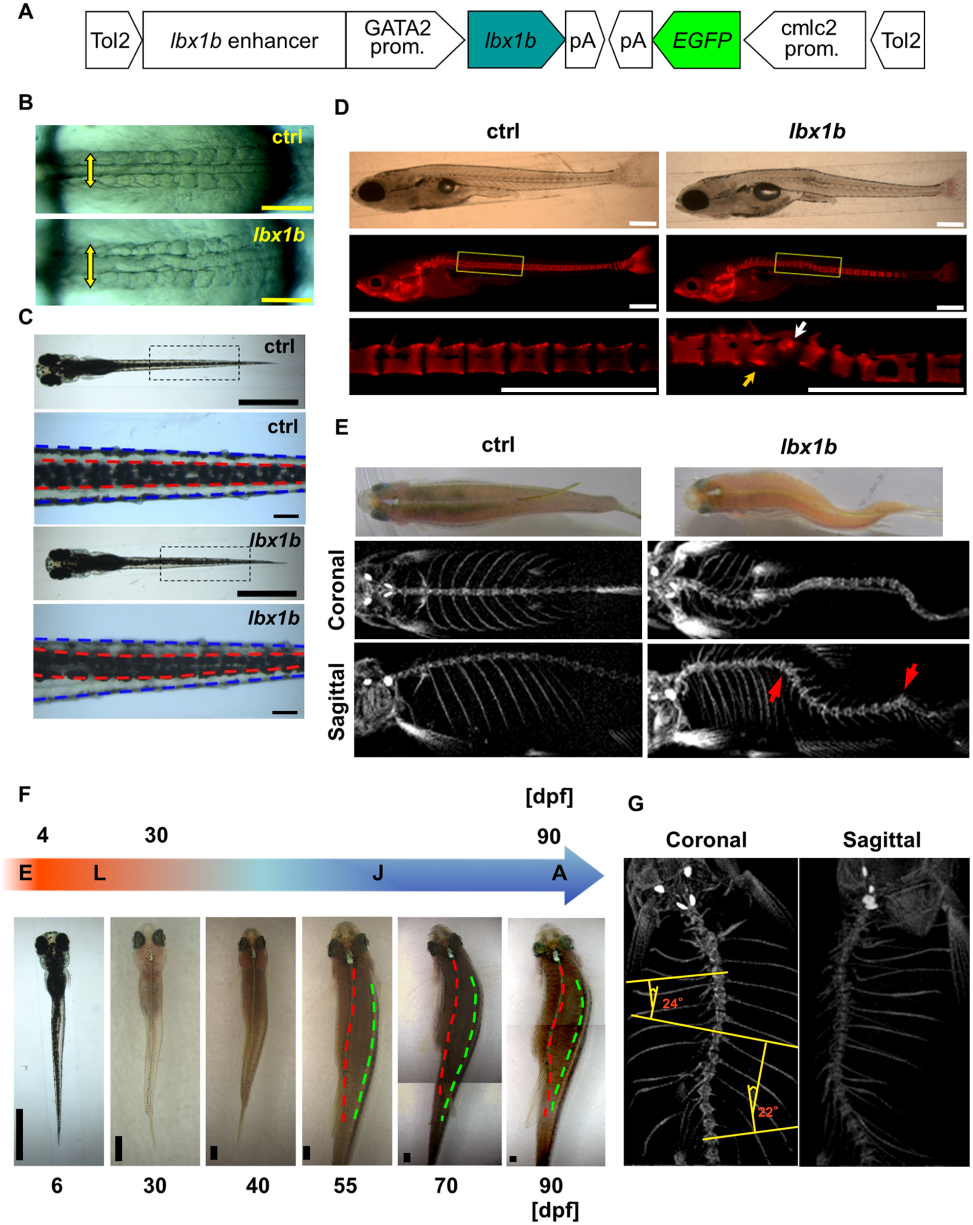
Scoliosis in transgenic founder zebrafish expressing *lbx1b* using the *GATA2* minimal promoter and the *lbx1b* enhancer. (A) The transgene construct is shown. The *GATA2* minimal promoter and *lbx1b* enhancer cooperatively drive *lbx1b* expression. (B) Dorsal views of live *GATA2-1b*:*MCS* (ctrl) or *GATA2-1b*:*lbx1b* (*lbx1b*) injected embryos with 10–13 somites. Somite arrangement is bilateral and symmetric in control embryos, but asymmetrical in *lbx1b* embryos. The yellow arrows indicate the mediolateral length of somites. (C) Dorsal views of 6 dpf larvae. The red and blue dotted lines indicate the boundaries of the dorsal melanophore stripes and the trunk, respectively. A displaced dorsal melanophore stripe was observed in *lbx1b* larvae. (D) Lateral views of alizarin red-stained larvae at 21 dpf. The white and yellow arrows indicate the hemivertebrae and block vertebra that developed from the deformed notochord in *lbx1b* larva, respectively. (E) Dorsal views and micro-computed tomography (μCT) analysis of adult zebrafish. Scoliosis was observed in adult fish grown from embryos with mild notochord deformities. The coronal and sagittal planes are reconstructed from μCT images of the fish in the upper panels, each showing a gross appearance. The red arrows indicate vertebral malformation. (F) Diagram of zebrafish growth stages. Continuous observation of one *GATA2*-*1b*:*lbx1b*-injected larva with a displaced dorsal melanophore stripe, but without notochord deformation, from 6 to 90 dpf. The red and green dotted lines indicate the dorsal middle lines and the right upper boundary of the lateral stripe, respectively. Progressive scoliosis with rotation of the longitudinal axis of the body was observed after 55 dpf. E, embryo; L, larva; J, juvenile; A, adult. (G) μCT analysis of the three-dimensional structure of the spine of the same zebrafish in (F) showing scoliosis. Cobb’s angle was measured in the coronal plane. Scale bars in (B): 200 μm; (C): 1 mm or 100 μm; (D): 500 μm; and (F): 1 mm.

To investigate the effects of *lbx1b* overexpression on endogenous expression domains during axial development, we forced *lbx1b* expression under the control of the previously characterized *lbx1b* enhancer [44] and the *GATA2* minimal promoter by microinjecting a *GATA2*-*1b*:*lbx1b* plasmid (Fig 8A). We confirmed that reporter expression driven by the regulatory elements in *Tg(GATA2-1b*:*EGFP*) generally recapitulated the endogenous expression of *lbx1b*, *lbx1a*, or *lbx2* at different developmental stages (S10 Fig). Similarly to embryos injected with mRNAs, many 48 hpf embryos injected with *GATA2*-*1b*:*lbx1b* developed severe body curvature (S11 Fig), abnormalities in somite morphology (Fig 8B), notochord deformity (S12A Fig), and a displaced dorsal melanophore stripe (Fig 8C). Some of the larvae with a displaced dorsal melanophore stripe had no apparent notochord deformity (S12B Fig). The majority of *Tg(GATA2-1b*:*lbx1b)* F1 embryos presented with a severe malformation and died within 24 hpf (S13 Fig). Some were alive at 48 hpf, developing serious axial body curvature, but died within 72 hpf (S13 Fig).

Unlike the F1 generation of *Tg(GATA2-1b*:*lbx1b)*, which is embryonic lethal, some founder *Tg(GATA2-1b*:*lbx1b)* with almost a straight trunk could survive to adulthood, thus allowing our observation of the later developmental stages in this model. We monitored embryos with a mild notochord deformity induced by injection of *GATA2*-*1b*:*lbx1b* (S12A Fig) (n = 41) until adulthood, together with wild-type siblings as controls (n = 45). Thirteen *Tg(GATA2-1b*:*lbx1b)* and two control zebrafish died within 21 days. The deformed notochord (S12A Fig red arrow) gradually ossified to form a spine, leading to vertebral malformations (n = 27, *p* < 0.01) such as hemivertebrae (Fig 8D, white arrow) and block vertebra (Fig 8D, yellow arrow) at the location of the notochord deformity. Eventually, these zebrafish showed scoliosis with vertebral malformations mimicking CS (Fig 8E and S1 video). No apparent spinal deformity was identified in the control (Fig 8B–8E). Thus, local notochord deformity in founder *Tg(GATA2-1b*:*lbx1b)* develops into CS-like scoliosis with vertebral malformations.

To investigate the possibility of AIS-mimicking scoliosis in *Tg(GATA2-1b*:*lbx1b)* during the period corresponding to human adolescence (Fig 8F), we kept transgenic larvae that had a displaced dorsal melanophore stripe without an apparent notochord deformity (n = 45), together with their wild-type siblings (n = 60). Eight *Tg(GATA2-1b*:*lbx1b)* and two control zebrafish died within 30 days. In 19 of the 37 surviving *Tg(GATA2-1b*:*lbx1b)* (*p* < 0.01), significant scoliosis, with rotation of the longitudinal body axis but without visible vertebral malformations, was observed by 55 dpf and then developed progressively until 90 dpf (Fig 8F, 8G and S2 video). Additionally, there was a significant female bias (16/19) for the prevalence of scoliosis (*p* < 0.01). No apparent spinal deformity was identified in the control group (0/58). These results indicate that mild body axis deformation resulting from the increased expression of *lbx1b* could cause irregular trunk development such as notochord deformity and a displaced dorsal melanophore stripe, further leading to the later development of CS- or AIS-like scoliosis.

## Discussion

We demonstrate here that the most significantly associated SNP, rs11190870 [15] could confer AIS susceptibility by activating *LBX1* transcription. Our gain-of-function approaches using the zebrafish model revealed that the elevated expression of human *LBX1* or any of the zebrafish genes *lbx1a*, *lbx1b*, and *lbx2* causes body axis deformation at various stages of embryonic and subsequent growth in zebrafish. Embryonic body curvature prior to vertebral column formation is associated with defective convergent extension involving the downregulation of *wnt5b* during gastrulation to disrupt axial development. Defective convergent extension and embryonic body curvature phenotypes were mostly rescued by the overexpression of *wnt5b* and *RhoA*, key molecules in the Wnt/PCP signaling pathway. An embryonic lethal phenotype could be alleviated by chimeric expression of *lbx1b* under the control of the *GATA2* minimal promoter and the *lbx1b* enhancer in larvae, thus allowing observation of the later onset of the spinal curvature with or without vertebral malformation in zebrafish. Thus, as a step towards better understanding of the genetic pathophysiology of scoliosis, our study provide a new evidence for a pathological role of *LBX1* and its zebrafish homologs in body axis deformation.

The most significant SNP associated with AIS (rs11190870) is located in the intergenic region [15]. The nearest gens are *LBX1* and *FLJ41350*, which are 7.5 kb upstream and 8.1 kb downstream of rs11190870, respectively. Using 3C assays, we found that the genome sequence surrounding rs11190870 physically interacts with the *LBX1* and *FLJ41350* promoters. In luciferase assays, significantly higher promoter activity was detected in the direction toward *LBX1*, but not toward *FLJ41350*. EMSAs revealed that some nuclear proteins bound specifically to the genome sequences around rs11190870 with higher affinity to the risk allele. Given that risk variants could disrupt or create a binding site for a transcription factor, any change of *LBX1* expression driven by the variants, including downregulation, upregulation, and alteration in temporospatial distribution, would be possible. Expression quantitative trait loci (eQTL) data are available only for peripheral blood cells, which showed no association between the *LBX1* expression level and the rs11190870 genotype (Human genetic variation database. (http://www.genome.med.kyoto-u.ac.jp/SnpDB/index.html). However, further studies on eQTL are hampered by a lack of information on which types of tissues or cells are relevant to AIS pathogenesis.

So far, phenotypes associated with CS and AIS have not been reported in *Lbx1* null mice and *lbx* gene knockdown morphants in zebrafish or *Xenopus* [25, 28–30]. Our *lbx1b* or *lbx2* single knockout zebrafish mutants generated by targeting the first exon using Platinum TALENs [45] also did not exhibit embryonic axial body curvature or scoliosis. The database of Zebrafish Mutation Project also shows that normal development is observed in *lbx1a* or *lbx2* nonsense mutants (https://www.sanger.ac.uk/sanger/Zebrafish_Zmpsearch/lbx1), although information on the associated phenotype of double or triple knockout zebrafish is not available. Previous studies demonstrated a dominant-negative effect by the removal of the engrailed domain from Xenopus Lbx1 that normally functions as a repressor [22, 46]. Injection of *lbx1aΔeh*, *lbx1bΔeh*, or *lbx2Δeh* mRNA did not cause any body curvature as shown in our study. Thus, the current data do not support the possibility that loss-of-function of *LBX1* is involved in susceptibility to scoliosis. In contrast, we found a significant increase of promoter activity in the presence of the genomic region with rs11190870 found in the risk allele. Considering that rs11190870 could confer AIS susceptibility by activating *LBX1* transcription, it would be reasonable to assume that upregulation of human *LBX1* may contribute to some aspects of the pathogenic mechanism in scoliosis.

The ladybird protein is a member of the homeobox transcription factor family with an engrailed repressor domain at the N-terminus [22]. Overexpression of *LBX1* and any one of *lbx1a*, *lbx1b*, *or lbx2* caused defective convergent extension movements that led to curvature of the body axis. Upon overexpression of the *lbx genes* without the engrailed repressor domain, body curvature was not observed in the embryos, suggesting that *Lbx* genes negatively regulate their target genes as repressors. Indeed, our *in vivo* luciferase assays revealed that *lbx1b* significantly represses the promoter activity of *wnt5b*. Hence, *lbx1b* downregulates *wnt5b* expression during gastrulation at the transcriptional level, thereby causing defective convergent extension followed by deformation of the body axis.

Both canonical and non-canonical Wnt signaling pathways are involved in convergent extension movements during gastrulation. A shortened-curled tail was reported in a *Wnt-5* mutant (*ppt*^*−/−*^) with defective convergent extension [47]. AIS- and CS-like scoliosis are also observed in zebrafish mutants of *ptk7*, which regulates both canonical and non-canonical Wnt signaling activity [37, 48]. The same group identified a novel sequence variant within a single IS patient that disrupted *PTK7*. In this study, we found that the elevated expression of *lbx1* in zebrafish evokes *wnt5b* downregulation, suggesting that aberrant Wnt/PCP signaling causes defective convergent extension in our experimental model. Interestingly enough, our approach investigating the etiology of scoliosis from the opposite direction also led to the hypothesis that a dysregulated Wnt signaling pathway is involved in both CS and IS pathogenesis.

Non-canonical Wnt/PCP signaling is involved in a variety of events independently of β-catenin [49]. During axis formation in vertebrates, the Wnt/PCP pathway regulates cell polarity and cell motility by modulating the activity of Rho family small GTPases. Especially, RhoA-ROCK signaling mainly acts downstream of *wnt5* and *wnt11* in zebrafish embryos [50]. Co-injection of mRNA for *wnt5b* or *RhoA* mRNA with *lbx1b* mRNA rescued defective convergent extension leading to embryonic body curvature. These findings strongly support our hypothesis that misregulation of Wnt/PCP signaling induced by *lbx1b* overexpression is responsible for defective convergent extension followed by body axis deformation.

To date, CS and IS have been considered not to be etiologically relevant, but it has been reported previously that a family history of IS was observed in 17.3% of 237 families with CS [51]. Another study of 31 CS cases also reported that three (10%) had first-degree relatives with IS [52]. The overlapping familial aggregates of CS and IS suggested the possibility of a common cause for these clinically distinct diseases. The uniform overexpression of *lbx1b* either ubiquitously or in the endogenous expression domain results in severe defective convergent extension leading to morphological defects in both mesoderm and ectoderm patterning followed by early death prior to notochord mineralization to form the spine. In contrast, mosaic expression of *lbx1b* under the control of the *lbx1b* enhancer in larvae alleviated the embryonic lethal phenotype with body curvature and thereby allowed the later onset of scoliosis with or without vertebral malformation in zebrafish. The F1 embryos generated by the AIS- and CS-like mosaic transgenic founders presented with severe body axis deformation including convergent extension defects and body curvature. Thus, our observations that the elevated expression of *lbx1b* causes both AIS- and CS-like scoliosis may provide a new perspective for the shared genetic basis of AIS and CS.

Some of the founder *Tg(GATA2*-*1b*:*lbx1b*) with a displaced dorsal melanophore stripe without apparent notochord deformity developed scoliosis with rotation around the longitudinal axis of the body, but without visible vertebral malformations. These results suggest that subtle deformities in the early body axis may be later accentuated during the growth spurt. In fact, AIS patients appear to be quite normal until adolescence. It is reasonable to postulate that an early event such as defective axial development resulting from the upregulation of *LBX1* may be too mild to be detected in potential AIS patients until the growth spurt. In a late-onset polygenic disease such as AIS, even such subtle abnormalities may be sufficient to accumulate growth irregularities and greatly aggravate biomechanical instability during adolescence in association with additional genetic or environmental factors. However, at present, considering that the *GATA2* minimal promoter and an *lbx1b* enhancer could drive *lbx1b* expression in neural tissue later in development [44], we cannot exclude the possibility that *lbx1b* expression after convergent extension causes idiopathic scoliosis. Thus, we need to determine carefully the mechanism by which *lbx1b* causes the AIS-like phenotype in the mosaic transgenic founders.

Polygenic diseases including AIS are triggered by the combination of a number of susceptibility genes whose individual contribution may be relatively small. It is also considered that these diseases could occur when a threshold of quantitatively-varying risk or liability influenced genetically and environmentally is exceeded [53]. Unlike a monogenic disease caused by a mutation in one gene, it appears that the cumulative effects combined with additional factors for a relatively long time lead to the onset of clinical manifestations of AIS, even though the contribution of each individual gene is small. Our study provides a new evidence for the possible involvement of *LBX1*-induced mild defects during embryonic axial development in AIS susceptibility. As the faithful recapitulation of the late-onset polygenic disease in the animal model has not been generally established yet, our current experimental approaches are still fraught with limitations. Further studies are necessary for establishment of a genetic animal model recapitulating the expression of *LBX1* in an analogous way to that in AIS patients.

## Materials and Methods

### Ethics statement

All of the animal experimental procedures used in this study were approved by the Animal Care Committee of the Institute for Frontier Medical Sciences, Kyoto University and conformed to institutional guidelines for the study of vertebrates

### Cell culture and 3C assay

Rhabdomyosarcoma cells and *A172* human glioblastoma *cells* (A172 cells) from ATCC and HeLa cells were obtained from the Japanese Collection of Research Bioresources Cell Bank (Osaka, Japan). The cells were maintained at 37°C under 5% CO_2_ in Dulbecco’s modified Eagle’s medium-high glucose supplemented with penicillin (50 U/mL), streptomycin (50 g/mL), and 10% fetal bovine serum. The cells were crosslinked with 37% formaldehyde solution at a final concentration of 1% in a 37°C dry incubator for 10 min, followed by an additional incubation at 4°C for 2 h. The crosslinked protein-chromatin material was purified by 8 M urea ultracentrifugation and digested with *Sau*3AI as described previously. A 2-g aliquot of chromatin was diluted in a ligation buffer and ligated with T4 DNA ligase (Fermentas) for 4 h. After reversing the crosslinks, the ligated DNA was amplified by PCR with various combinations of primers using GoTaq Hot Start Master Mix (Promega).

### Electrophoretic mobility shift assay

We prepared nuclear extracts from rhabdomyosarcoma and A172 cells as described previously [54]. We prepared probes for the risk (R) and non-risk (N) alleles of rs11190870 by annealing 17-bp complementary oligonucleotides and labeling with digoxigenin (DIG)-11-ddUTP (Roche). For competition experiments, nuclear extracts were pre-incubated with excess unlabeled probes. We detected DNA-protein complexes using a DIG gel shift kit according to the manufacturer’s instructions (Roche).

### Construction of plasmids, transfection, and luciferase reporter assay

We amplified the *LBX1* promoter fragment (-917 to +153) in both directions by PCR and cloned them into the pGL4.10 promoter-less luciferase reporter vector (Promega). The constructs were co-transfected with the pGL4.73 Renilla luciferase vector (hRluc/SV40) as an internal control. Transfection of each construct was performed using TransIT-LT1 (Mirus Bio LLC). HEK 293T cells were maintained at 37°C under 5% CO_2_ in Dulbecco’s modified Eagle’s medium-high glucose supplemented with 10% fetal bovine serum. Transfection was performed with Lipofectamine LTX and PLUS reagent (Life Technologies). After 24 h of transfection, the cells were harvested and luciferase activity was measured using a Pick&gene dual luciferase detection kit (Toyo B-Net Co.).

### Zebrafish

The RIKEN Wako (RW) strain and AB strain were obtained from the Zebrafish National BioResource Center of Japan (http://www.shigen.nig.ac.jp/zebra/) and Kondoh ERATO Laboratory, respectively. Adult fish were maintained under a 14 h light–10 h dark cycle at 28°C. Embryos were kept at 28°C and staged by hpf or dpf [55]. The RW strain was subjected to micro-injection and whole-mount *in situ* hybridization. The AB strain was used for the preparation of total RNA. The established line *Tg(UAS*:*EGFP)* [41] was generously provided by Dr. Koichi Kawakami (National Institute of Genetics).

### *In vitro* synthesis and microinjection of mRNA

Specific primers for zebrafish *lbx1a* (NM_001025532), *lbx1b* (NM_001163312), and *lbx2* (NM_001007134), and human *LBX1* (NM_006562.4), *FLJ41350* (NR_029380), and *RhoA* (NM_001664.2) were designed based on the nucleotide sequences from GenBank (S1 Table). The cDNAs were amplified by PCR from a cDNA library and cloned into the *pCS2*(*+)* vector. Deletion constructs of the engrailed domain and homeodomain of *lbx1* were generated by inverse PCR. Capped mRNAs were synthesized using an SP6 RNA polymerase *in vitro* transcription kit (Life Technologies) and purified using a MEGAclear Kit (Life Technologies) according to the manufacturer’s instructions. A mixture containing 50/100/150 pg mRNA for *lbx1a*, *lbx1b*, *lbx2*, *LBX1*, and *FLJ41350*, 15 pg mRNA for *RhoA*, and 40 pg mRNA for *wnt5b* and *RAC1* was injected into the cytoplasm of one-cell-stage embryos.

### Construction of TALENs

Highly active Platinum TALENs were constructed using two-step Golden Gate assembly method as described previously with a slight modification [45]. DNA-binding modules were assembled with the two-step Golden Gate method using the Platinum Gate TALEN Kit (Addgene, Kit #1000000043). pCS2-based vectors were used as destination vectors. The target sequence was 5’-TAAACCCCCTGGACCACcttccaccacccgcgAGCTCCAACAA GCCCTTA-3’, where uppercase and lowercase letters indicate *lbx1b* TALEN recognition sequence and spacer sequence, respectively. We found polymorphism of RW WT in the left TALE-binding sequence; TAAACCCCCTGGACCAC and TGAATCCCCTGGACCAC, both of which are silent mutations. The target sequence was 5’-TTGCAGTCCAGCGGCGAG gagaggcggcggggtCCCTTGGACCAACTCCCA-3’, where uppercase and lowercase letters indicate *lbx2* TALEN recognition sequence and spacer sequence, respectively.

### Generation of *lbx1b* mutant

Genomic DNA was extracted from the caudal fins of *lbx1b* TALENs mRNA-injected zebrafish. For sequencing, PCR products were amplified from the genomic DNA and phosphorylated by T4PNK (TAKARA BIO INC.), and then subcloned into EcoRV site of pBluescript II SK(+) vector. We identified F0 fishes carrying multiple mutations in the target site, and then generated and screened a F1 fish with a nonsense mutation by crossing the F0 and wild type fishes. *lbx1b*^+/-^ and *lbx2*^+/-^ mutant were generated by crossing F1 and wild type fishes. *lbx1b*^-/-^ and *lbx2*^-/-^ were further generated by intercrossing *lbx1b*^+/-^ and *lbx2*^+/-^, respectively.

### Generation of transgenic zebrafish

The *Tol2* transposon/transposase system [41, 56–59] was employed for the establishment of transgenic zebrafish. The coding sequence of *lbx1b* was cloned into *pME-MCS* to generate *pME-lbx1b*. The complementary sequence of the E1b promoter, *EGFP*, and polyA were cloned into *p5E-UAS-E1b* to generate *p5E-polyA-EGFP-E1b-UAS-E1b*. The driver construct (Fig 3A) was generated by recombining *p5E-hsp70I*, *pME-Gal4VP16*, *p3E-polyA*, and *pDestTol2CG2* with Gateway LR Clonase II Enzyme mix (Life Technologies). Similarly, the responder construct (Fig 3A) was generated by recombining *p5E-polyA-EGFP-E1b*-*UAS-E1b*, *pME-lbx1b*, *p3E-polyA*, and *pDestTol2CG2*. Capped mRNA of medaka *Tol2* transposase was prepared by *in vitro* translation as described above. A mixture containing 50 pg transposase mRNA and 40 pg *Tol2* transgenic plasmid was injected into the cytoplasm of one-cell-stage embryos. F1 fish were acquired by outcrossing EGFP-positive F0 with RW fish, and screened by cardiac fluorescence. F2 lines were then generated by outcrossing F1 and RW fish. All kept F2 lines yielded about 50% EGFP-positive progeny when mated to RW fish, which suggested there was a single Tol2 insertion site. The *lbx1b* enhancer located from +1316 to +2383 bp downstream of the *lbx1b* transcription start site [44] was cloned into *pME-MCS*. The enhancer activity *in vivo* was confirmed using zebrafish enhancer detection (ZED) [60]. To generate *p5E-lbx1b* enhancer-*GATA2*, the 2.3-kb *Bam*HI fragment from the ZED-*lbx1b* enhancer, was cloned into the *Bam*HI site of *p5E-MCS*. The constructs *GATA2*-*1b*:*lbx1b* or *GATA2*-*1b*:*MCS* were generated by recombining *p5E-lbx1b* enhance-*GATA2*, *pME-lbx1b or pME-MCS*, *p3E-polyA*, and *pDestTol2CG2*.

### Heat shock treatment

Embryos at 4 hpf or 12 hpf in E3 buffer were placed on block incubator and heated up to 38°C gradually, and then maintained at 38°C for 30 min. After heat shock treatment, they were gradually cooled to 28.5°C. The heat shock treatment causes neither an anomaly nor a decrease in viability.

### Western blot analysis

Larvae were homogenized with a Dounce tissue grinder and lysed with lysis buffer (50 mM Tris-HCl [pH 7.5], 150 mM NaCl, 1% NP-40, 0.1% SDS) containing protease inhibitor cocktail (Roche). Lysates were mixed with 5× Laemmli sampling buffer containing 100 mM DTT and boiled at 95°C for 3 min. Proteins were separated by SDS-PAGE and transferred onto PVDF membranes (Merck Millipore). After blocking with BLOCKING ONE (Nacalai Tesque), the membranes were incubated with primary antibodies in phosphate-buffered saline containing 0.1% Tween 20 and 10% BLOCKING ONE, followed by incubation with horseradish peroxidase-conjugated secondary antibodies. Signals were detected with SuperSignal West Pico Chemiluminescent Substrate (Thermo Scientific) and images were captured by ImageQuanta LAS 4000 (GE Healthcare Bio-Sciences).

### Whole-mount *in situ* hybridization

Whole-mount *in situ* hybridization was performed as described previously [61]. Sense and antisense riboprobes for *hgg1*, *dlx3b*, *ntl*, *papc*, *uncx4*, *wnt5b*, *wnt11* [42], *chd* and *vox* [48] were generated by *in vitro* translation using a digoxigenin (DIG) RNA labeling kit with T7 or T3 RNA polymerase (Roche). Hybridization signals were detected with an alkaline phosphatase-conjugated anti-DIG antibody (Roche) according to the manufacturer’s instructions. For quantification, the image colors of *in situ* hybridization were inverted, and Area, Integrated Density, and Mean Gray Value were measured by ImageJ. The corrected Gray Value = Integrated Density − (Area of the selected embryos × Mean Gray Value of background readings).

### RNA extraction and quantitative RT—PCR

Total RNA was extracted from zebrafish embryos injected with *lbx1b* mRNA using an RNeasy Plus Mini kit (QIAGEN). Two hundred nanograms of total RNA were used to synthesize cDNA with a PrimeScript RT reagent Kit (Takara Bio). Quantitative RT-PCR was performed using SYBR Premix Ex Taq II (Takara Bio) on a StepOne instrument (Life Technologies). Relative mRNA expression was normalized to *ef-1α* and calculated using the 2^−ΔΔCT^ method. Specific primers for quantitative RT—PCR are listed in S1 Table.

### *In vivo* luciferase assay

A 569-bp insulator of chicken *β-globin* (*BGI*) and firefly luciferase (*luc*) were amplified from the ZED vector and pGL3-basic vector (Promega), respectively. The resultant amplification products were cloned into *pME-MCS* to construct the promoter-less *pME-BGI-luc* plasmid. Two fragments of approximately 2 kb upstream of each transcription start site of *wnt5b* were amplified from zebrafish genome DNA with the primers listed in S1 Table. These were cloned into *pME-BGI-luc*, *pME-BGI-P1-luc* plasmid (P1), and *pME-BGI-P2-luc* (P2) by the In-Fusion technique (Clontech). A mixture containing fluorescein isothiocyanate (FITC)-dextran (SIGMA) and luciferase plasmids with or without *lbx1b* mRNA was injected into one-cell-stage embryos. FITC fluorescence intensity was quantified using a fluorescence microscope (Leica MZ 16 FA) and ImageJ software. Embryos were then lysed individually and luciferase activity was measured as described previously [62]. The measured activity was normalized by the FITC fluorescence intensity of an individual embryo.

### Skeletal preparations

Bones in fixed larvae were stained with alizarin red (Wako).

### Skeletal imaging by micro-computed tomography analysis

Vertebral bone morphology of adult zebrafish was analyzed by micro-computed tomography scans with inspeXio SMX-90CT (SHIMADZU). Three-dimensional reconstruction and videos were generated with ImageJ software.

### Phenotype scoring and statistical analysis

Embryos were examined and scored for relevant phenotypes. Statistical analysis (SPSS 16.0) was performed by chi-square analysis for enumeration data and independent-samples *t* test or one-way ANOVA for measurement data to calculate *p* values under various conditions. Spearman’s correlation between relative fluorescence intensity and body curvature severity was calculated. A linear regression equation was calculated with SPSS.

## Supporting Information

S1 Fig Nucleotide sequence and deduced amino acid sequence of FLJ41350. Nucleotide sequences were determined by Sanger sequencing of RT-PCR and rapid amplification of cDNA ends (RACE) products. Nucleotides are numbered on the right. The initiation codon is underlined. It conformed to the Kozak sequence. The stop codon is indicated by an asterisk and a putative poly-adenylation signal is enclosed in an open box. Multiple transcription start sites (TSSs) clustered in a region of a few dozen base pairs were identified by 5′-RACE, but only the most major TSS is shown.
(TIF)

S2 Fig Local deformation of the notochord, displaced dorsal melanophore stripe, and ophthalmic abnormity caused by injection of *lbx1b* mRNA. Dorsal views of larvae at 6 dpf. The deformed notochord, displaced dorsal melanophore stripe, and anophthalmia in *lbx1b* mRNA-injected zebrafish are indicated by a green, yellow, and red arrow, respectively. Scale bar: 1 mm.
(TIF)

S3 Fig TALEN-induced *lbx1b* mutant. (A) Alignment of representative sequences of the PCR amplicons from *lbx1b* transcription activator-like effector nuclease (TALEN) mRNA-injected embryos showing insertions and deletions. TALEN-binding sites in exon 1 of *lbx1b* are indicated in red. Indels were detected in 32.7% (17/52) of clones from embryos injected with TALEN mRNA. A dash indicates a single nucleotide deletion. Inserted nucleotides are underlined. (B) DNA chromatographs for sequences of a wild-type (WT) and an established *lbx1b* nonsense mutant line. Three nucleotides (CAC) in WT were deleted and/or replaced with two nucleotides (TT) (indicated in red). (C) Schematic diagram showing a premature stop site caused by a TALEN-induced frameshift mutation in the first exon of *lbx1b*. Altered amino acids in the mutant are indicated in red. E1: exon 1; E2: exon 2; Eh, engrailed homology domain; Hd: homeobox domain.
(TIF)

S4 Fig TALEN-induced *lbx2* mutant. (A) Alignment of representative sequences of the PCR amplicons from *lbx2* transcription activator-like effector nuclease (TALEN) mRNA-injected embryos showing insertions and deletions. TALEN-binding sites in exon 1 of *lbx2* are indicated in red. Indels were detected in 40.9% (56/137) of clones from embryos injected with TALEN mRNA. A dash indicates a single nucleotide deletion. Inserted nucleotides are underlined. (B) DNA chromatographs for sequences of a wild-type (WT) and an established *lbx2* nonsense mutant line. Sixteen nucleotides were deleted and/or replaced with 32 nucleotides in the mutant (indicated in red). (C) Schematic diagram showing a premature stop site caused by a TALEN-induced frameshift mutation in the first exon of *lbx2*. Altered amino acids in the mutant are indicated in red. E1: exon 1; E2: exon 2; Eh, engrailed homology domain; Hd: homeobox domain.
(TIF)

S5 Fig Gal4/UAS-based bidirectional expression system. (A) The constructs of the Gal4/UAS-based bidirectional expression system. Heat shock (HS) treatment activates the *hsp70I* promoter in the driver construct (Driver) to express *Gal4-VP16*. Gal4-VP16 protein binds to the UAS on the responder construct (Responder) and activates the expression of *mCherry* and *EGFP* via E1b minimal promoters. (B) A positive correlation of expression level between the two genes flanking the UAS in *Tg(hsp*:*Gal4-VP*: *EGFP*:*UAS*:*mcherry*). The scale bar represents 1 mm.
(TIF)

S6 Fig Normal dorsoventral patterning in embryos with *lbx1b* overexpression. (A) Whole-mount *in situ* hybridization (WISH) for dorsal organizer gene *chd* and ventral gene *vox* in shield-stage embryos injected with buffer as control (ctrl) or *lbx1b* mRNA (*lbx1b*). Photos are taken from the animal pole side of embryos with dorsal to the left. (B) Quantitative analysis of the WISH signals shown in A. For quantification of the dorsoventral position, the angle **θ** [degree] shown in the panel A as measured. No significant change was observed for both *chd* (*p* = 0.261) and *vox* (*p* = 0.071) expression pattern. The numbers of control and *lbx1b* zebrafish embryos were 22 and 23 for *chd*, 23 and 23 for *vox*, respectively.
(TIF)

S7 Fig Quantification of *wnt5b or wnt11 expression*. Quantitative analysis of the signal intensity of *in situ* hybridization for *wnt5b* or *wnt11* by processing the images shown in Fig 6A. Significant differences (*p < 0.01) were observed in *wnt5b*.
(TIF)

S8 Fig Failure to rescue defective convergent extension and body curvature in *lbx1b*-overexpressing embryos by *Rac1* mRNA injection. (A) Dorsal view of *dlx3b/hgg1/ntl* expression in the tail bud of embryos injected with buffer (ctrl), *lbx1b mRNA* (*lbx1b*), or *lbx1b* and human *RAC1* mRNA (*lbx1b+Rac1*). (B) Quantitative analysis of convergent extension (CE) movement with embryos in (A) (ctrl, n = 15; *lbx1b*, n = 15; *lbx1b+Rac1*, n = 13). The extent of defective CE was evaluated by measuring the distance between the inner edges of bilateral *dlx3b* expression at 3 regions as indicated by the arrows in panel A. *RAC1* mRNA injection failed to rescue defective CE. *p < 0.01, NS: not significant. (C) Dorsal view of 48 hpf *Tg(hsp*:*Gal-VP; EGFP*:*UAS*:*lbx1b)* embryos upon heat shock at 4 hpf with buffer (*lbx1b*) or *RAC1* mRNA (*lbx1b*+*Rac1*) injection. (D) Quantitative analysis of body curvature with the embryos in (C) (*lbx1b*, n = 11; *lbx1b*+*Rac1*, n = 11). No significant change of the severity of body curvature was observed in *RAC1* mRNA-injected embryos. NS: not significant. Severity of body curvature was quantified by the angle as described in Fig 3. F2 lines of driver and responder transgenic fish were used. The dosages for embryo injections were 50 pg/embryo for *lbx1b* mRNA and 40 pg/embryo for *RAC1* mRNA.
(TIF)

S9 Fig Effect of *RhoA* mRNA injection on body curvature of *Tg(hsp*:*Gal-VP*:*EGFP*:*UAS*:*lbx1b)* with heat shock at 12 hpf. (A) Dorsal views of 48 hpf *Tg(hsp*:*Gal-VP*:*EGFP*:*UAS*:*lbx1b)* embryos upon heat shock (HS) at 12 hpf with buffer (HS12h) or *RhoA* mRNA (HS12h+*RhoA*) injection. (B) Quantitative analysis of body curvature in *Tg(hsp*:*Gal-VP*:*EGFP*:*UAS*:*lbx1b)* embryos upon HS at 12 hpf with buffer injection (*lbx1b*, n = 13) or *RhoA* mRNA injection (*lbx1b*+*RhoA*, n = 13). Significant change in axis development was not observed in *RhoA*-injected embryos with HS at 12 hpf. Severity of body curvature was quantified by the angle as shown in Fig 3E. F2 lines of driver and responder transgenic fish were used.
(TIF)

S10 Fig Characterization of an *lbx1b* enhancer in *Tg(GATA2-1b*:*EGFP*). (A) Construction of the transgene. The *GATA2* minimal promoter and an *lbx1b* enhancer cooperatively drive the expression of *EGFP*. The cardiac specific promoter cmlc2 drives EGFP expression in the heart as a transgenic marker. (B–D) Comparison of *EGFP* fluorescence with *lbx1a*, *lbx1b*, and *lbx2* expression in lateral views at the 90% epiboly-bud stage (B), 13–15 somites stage (C), and 24–26 somites stage (D).
(TIF)

S11 Fig Body curvature in embryos with *lbx1b* expressed under the control of the *GATA2* minimal promoter and an *lbx1b* enhancer. (A) Dorsal views of live embryos at 48 hpf. Body curvature was observed in zebrafish injected with *GATA2*-*1b*:*lbx1b* (*lbx1b*), but not in those injected with *GATA2*-*1b*:MCS (ctrl). The scale bar represents 500 μm. (B) Quantitative analysis of body curvature (cur) in 48 hpf embryos. The incidence of body curvature was significantly increased in *lbx1b* embryos (ctrl, 5%, n = 83; *lbx1b*, 46%, n = 94. *p* < 0.01). Scale bar in (A): 500 μm.
(TIF)

S12 Fig Notochord deformity and displaced dorsal melanophore stripe in embryos with *lbx1b* expressed under the control of the *GATA2* minimal promoter and an *lbx1b* enhancer. (A) Lateral views of embryos (48 hpf) injected with *GATA2*-*1b*:*mCherry* (ctrl) or *GATA2*-*1b*:*lbx1b* (*lbx1b*). Local notochord deformation (red arrow) was observed in *lbx1b* embryos. The lower panels show magnified views of the areas indicated by the dotted boxes in the corresponding middle panels. Scale bars represent 500 μm. (B) Quantitative analysis of the phenotypes of notochord deformation (nc+) and displaced dorsal melanophore stripe (str+) in 6 dpf zebrafish (ctrl, n = 224; *lbx1b*, n = 288).
(TIF)

S13 Fig Severe convergent extension defects and body curvature in *Tg(GATA2-1b*:*lbx1b)* F1 embryos. (A) The constructs used for transgenesis in zebrafish with the tol2 transposon system are shown. The cardiac specific promoter cmlc2 drives EGFP expression in the heart as a transgenic marker. (B) RT-PCR for 11 hpf embryos of *Tg(GATA2-1b*:*MCS)* F1 (MCS) and *Tg(GATA2-1b*:*lbx1b)* F1 (*lbx1b*) shows the elevated expression of *lbx1b* in *lbx1b* embryos. Ef-1α was used as a constitutive control. (C) Dorsal views of *In situ* hybridization for *papc* (paraxial mesoderm marker) at 11 hpf. Severe convergent defects were found in *lbx1b* embryos. The scale bar represents 200 μm. (D) Dorsal views of embryos at 48 hpf. Severe body curvature was observed in *lbx1b* embryos. The scale bar represents 1 mm.
(TIF)

S1 Table Primers for plasmid construction. (DOC)

S1 Video Micro-computed tomography of zebrafish with CS-like scoliosis. (AVI)

S2 Video Micro-computed tomography of zebrafish with AIS-like scoliosis. (AVI)
