# Supplementary figures and images for: Functional Investigation of a Non-coding Variant Associated with Adolescent Idiopathic Scoliosis in Zebrafish: Elevated Expression of the Ladybird Homeobox Gene Causes Body Axis Deformation
Source: PLoS Genet. 2016 Jan 28;12(1):e1005802. doi: 10.1371/journal.pgen.1005802 (PMC4731154; doi:10.1371/journal.pgen.1005802)

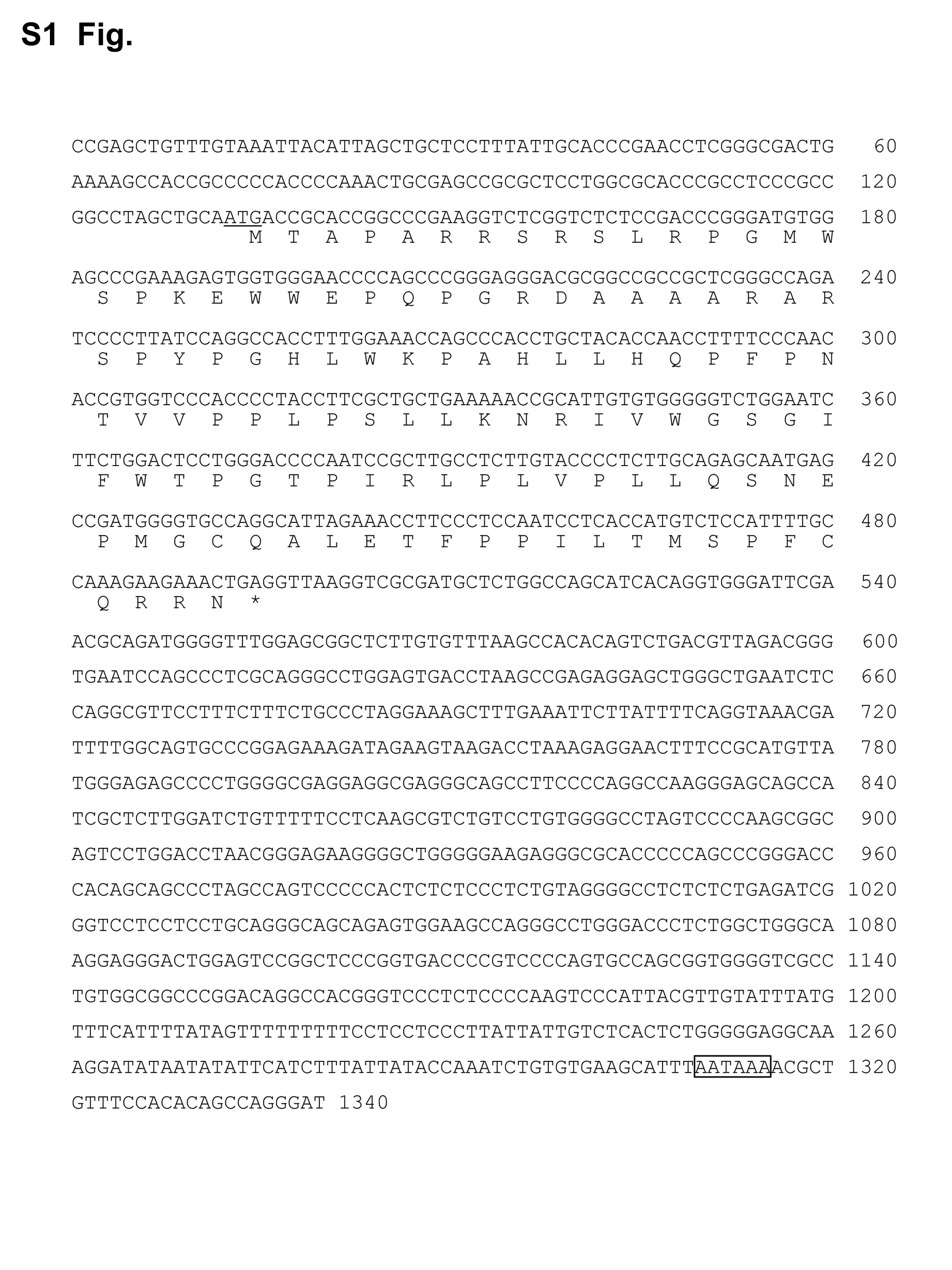

Supplement: S1 Fig — Nucleotide sequences were determined by Sanger sequencing of RT-PCR and rapid amplification of cDNA ends (RACE) products. Nucleotides are numbered on the right. The initiation codon is underlined. It conformed to the Kozak sequence. The stop codon is indicated by an asterisk and a putative poly-adenylation signal is enclosed in an open box. Multiple transcription start sites (TSSs) clustered in a region of a few dozen base pairs were identified by 5′-RACE, but only the most major TSS is shown. (TIF) [file pgen.1005802.s001.TIF]

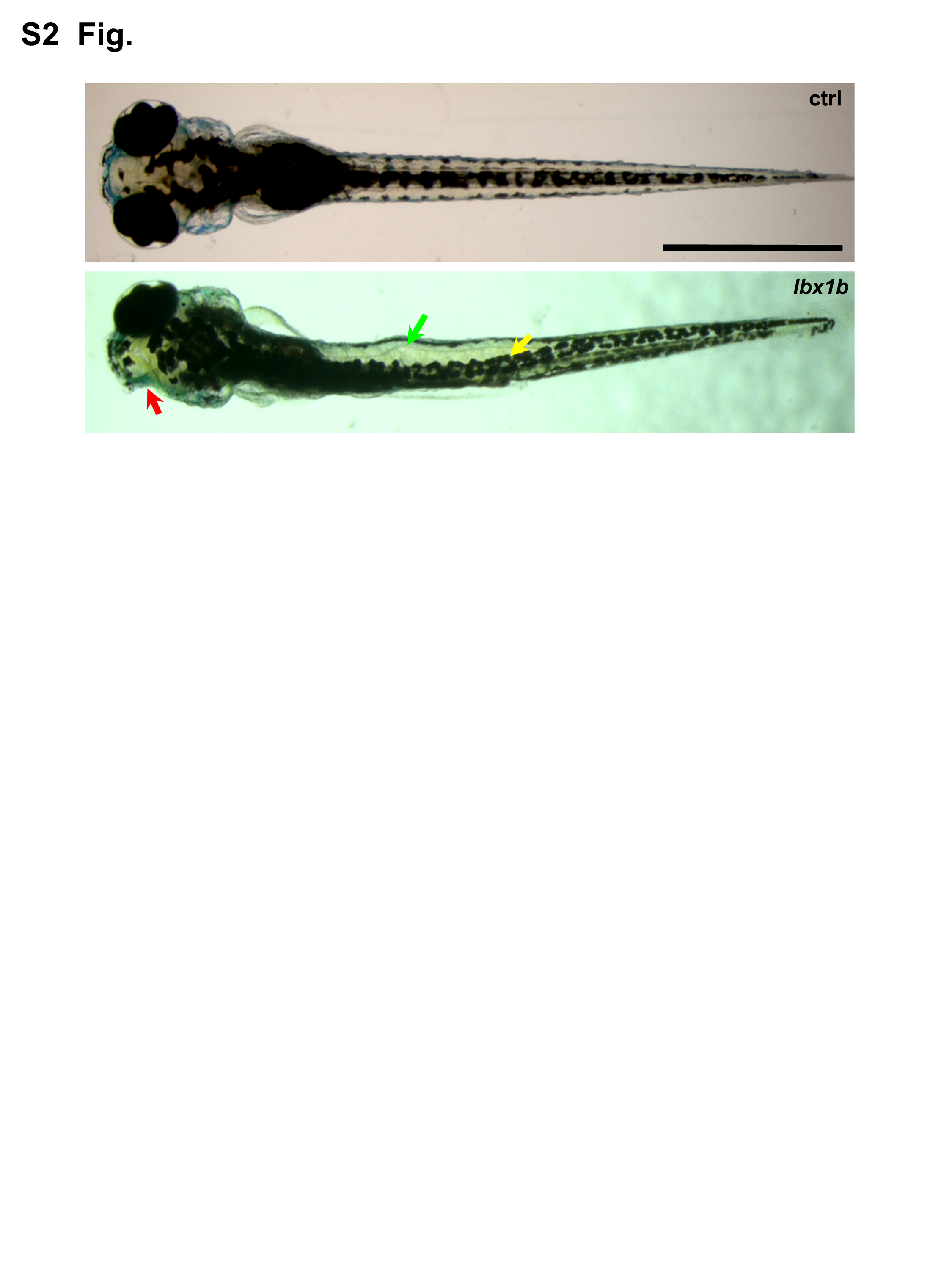

Supplement: S2 Fig — Dorsal views of larvae at 6 dpf. The deformed notochord, displaced dorsal melanophore stripe, and anophthalmia in lbx1b mRNA-injected zebrafish are indicated by a green, yellow, and red arrow, respectively. Scale bar: 1 mm. (TIF) [file pgen.1005802.s002.TIF]

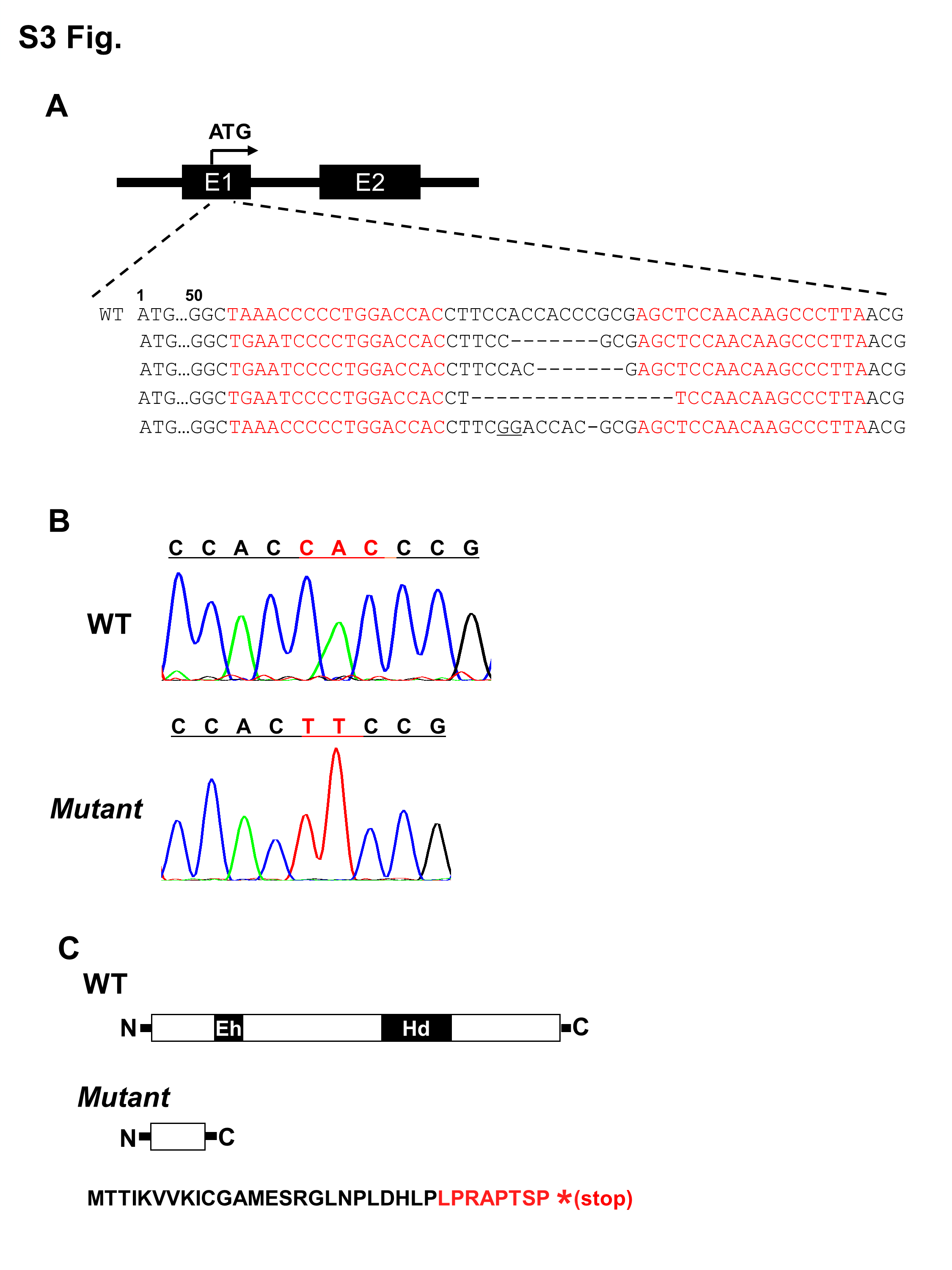

Supplement: S3 Fig — (A) Alignment of representative sequences of the PCR amplicons from lbx1b transcription activator-like effector nuclease (TALEN) mRNA-injected embryos showing insertions and deletions. TALEN-binding sites in exon 1 of lbx1b are indicated in red. Indels were detected in 32.7% (17/52) of clones from embryos injected with TALEN mRNA. A dash indicates a single nucleotide deletion. Inserted nucleotides are underlined. (B) DNA chromatographs for sequences of a wild-type (WT) and an established lbx1b nonsense mutant line. Three nucleotides (CAC) in WT were deleted and/or replaced with two nucleotides (TT) (indicated in red). (C) Schematic diagram showing a premature stop site caused by a TALEN-induced frameshift mutation in the first exon of lbx1b. Altered amino acids in the mutant are indicated in red. E1: exon 1; E2: exon 2; Eh, engrailed homology domain; Hd: homeobox domain. (TIF) [file pgen.1005802.s003.TIF]

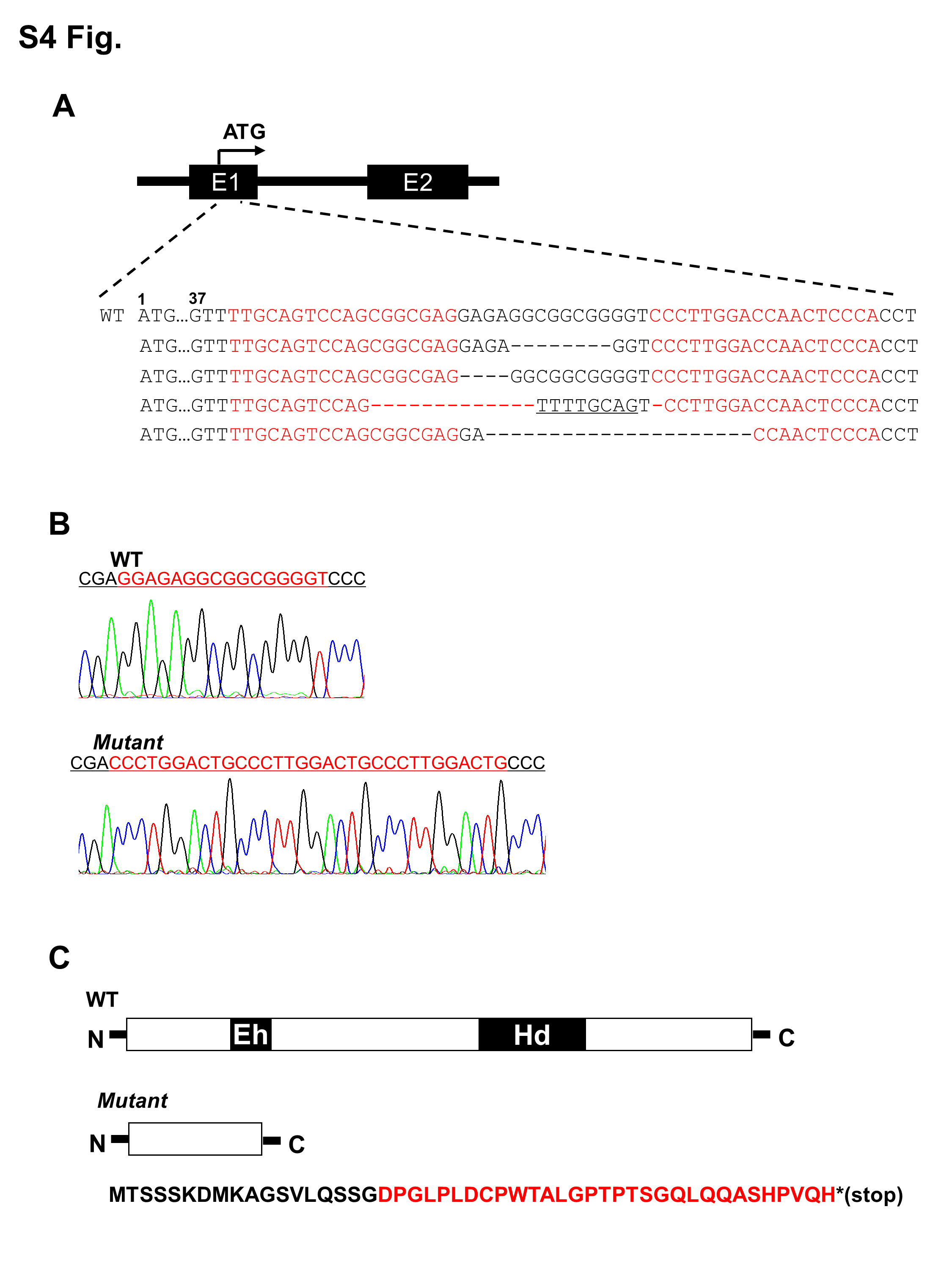

Supplement: S4 Fig — (A) Alignment of representative sequences of the PCR amplicons from lbx2 transcription activator-like effector nuclease (TALEN) mRNA-injected embryos showing insertions and deletions. TALEN-binding sites in exon 1 of lbx2 are indicated in red. Indels were detected in 40.9% (56/137) of clones from embryos injected with TALEN mRNA. A dash indicates a single nucleotide deletion. Inserted nucleotides are underlined. (B) DNA chromatographs for sequences of a wild-type (WT) and an established lbx2 nonsense mutant line. Sixteen nucleotides were deleted and/or replaced with 32 nucleotides in the mutant (indicated in red). (C) Schematic diagram showing a premature stop site caused by a TALEN-induced frameshift mutation in the first exon of lbx2. Altered amino acids in the mutant are indicated in red. E1: exon 1; E2: exon 2; Eh, engrailed homology domain; Hd: homeobox domain. (TIF) [file pgen.1005802.s004.TIF]

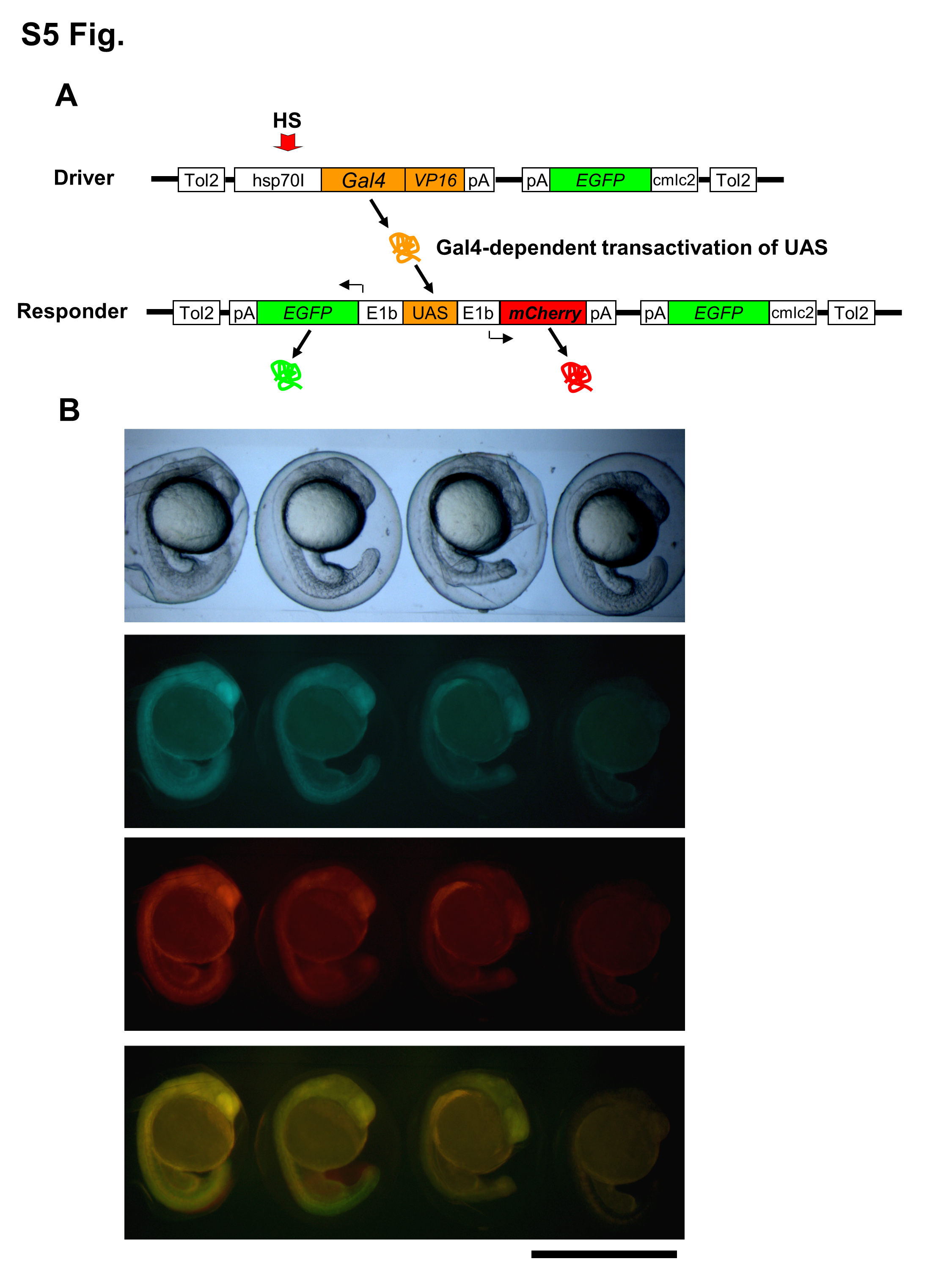

Supplement: S5 Fig — (A) The constructs of the Gal4/UAS-based bidirectional expression system. Heat shock (HS) treatment activates the hsp70I promoter in the driver construct (Driver) to express Gal4-VP16. Gal4-VP16 protein binds to the UAS on the responder construct (Responder) and activates the expression of mCherry and EGFP via E1b minimal promoters. (B) A positive correlation of expression level between the two genes flanking the UAS in Tg(hsp:Gal4-VP: EGFP:UAS:mcherry). The scale bar represents 1 mm. (TIF) [file pgen.1005802.s005.TIF]

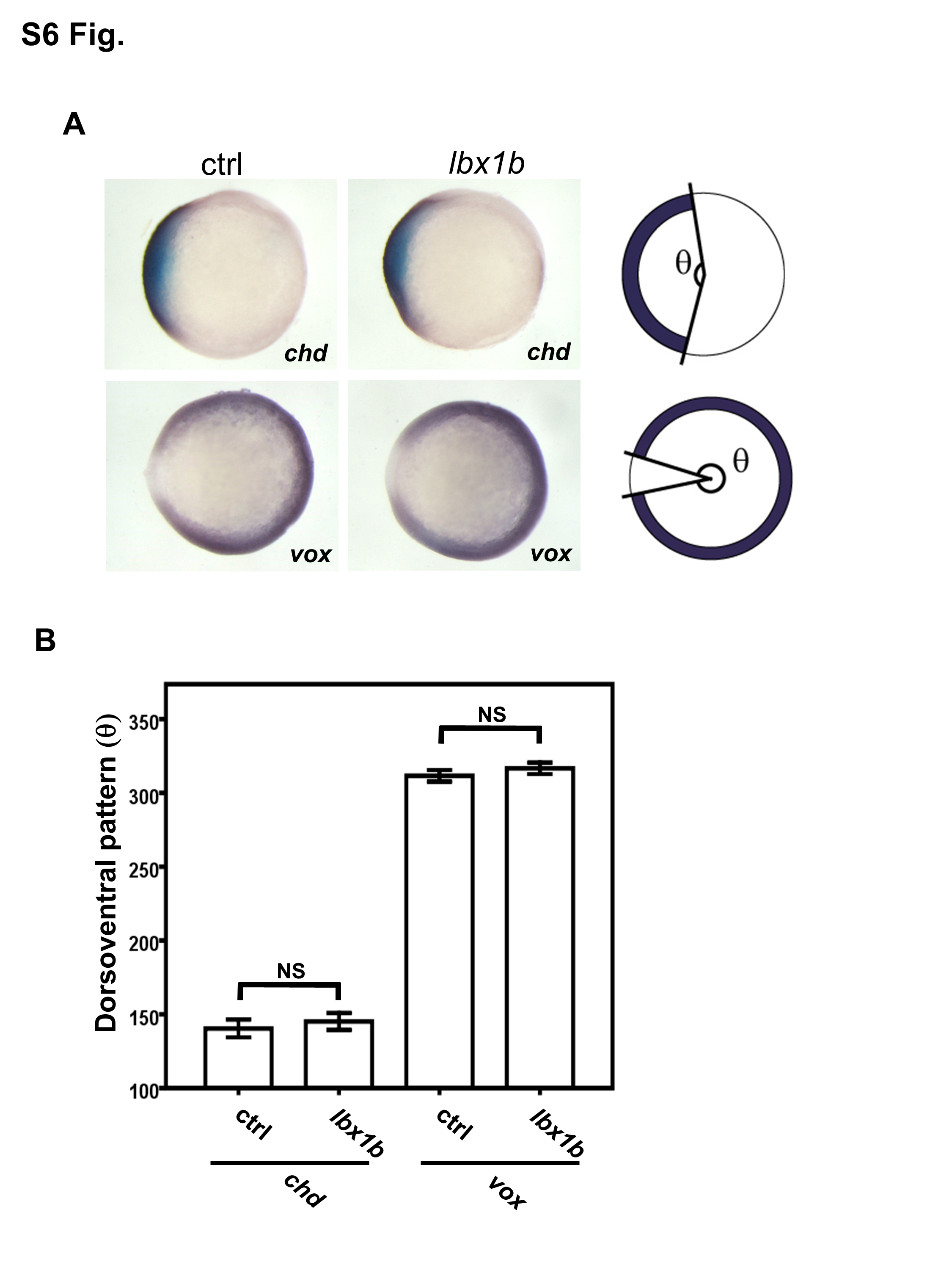

Supplement: S6 Fig — (A) Whole-mount in situ hybridization (WISH) for dorsal organizer gene chd and ventral gene vox in shield-stage embryos injected with buffer as control (ctrl) or lbx1b mRNA (lbx1b). Photos are taken from the animal pole side of embryos with dorsal to the left. (B) Quantitative analysis of the WISH signals shown in A. For quantification of the dorsoventral position, the angle θ [degree] shown in the panel A as measured. No significant change was observed for both chd (p = 0.261) and vox (p = 0.071) expression pattern. The numbers of control and lbx1b zebrafish embryos were 22 and 23 for chd, 23 and 23 for vox, respectively. (TIF) [file pgen.1005802.s006.TIF]

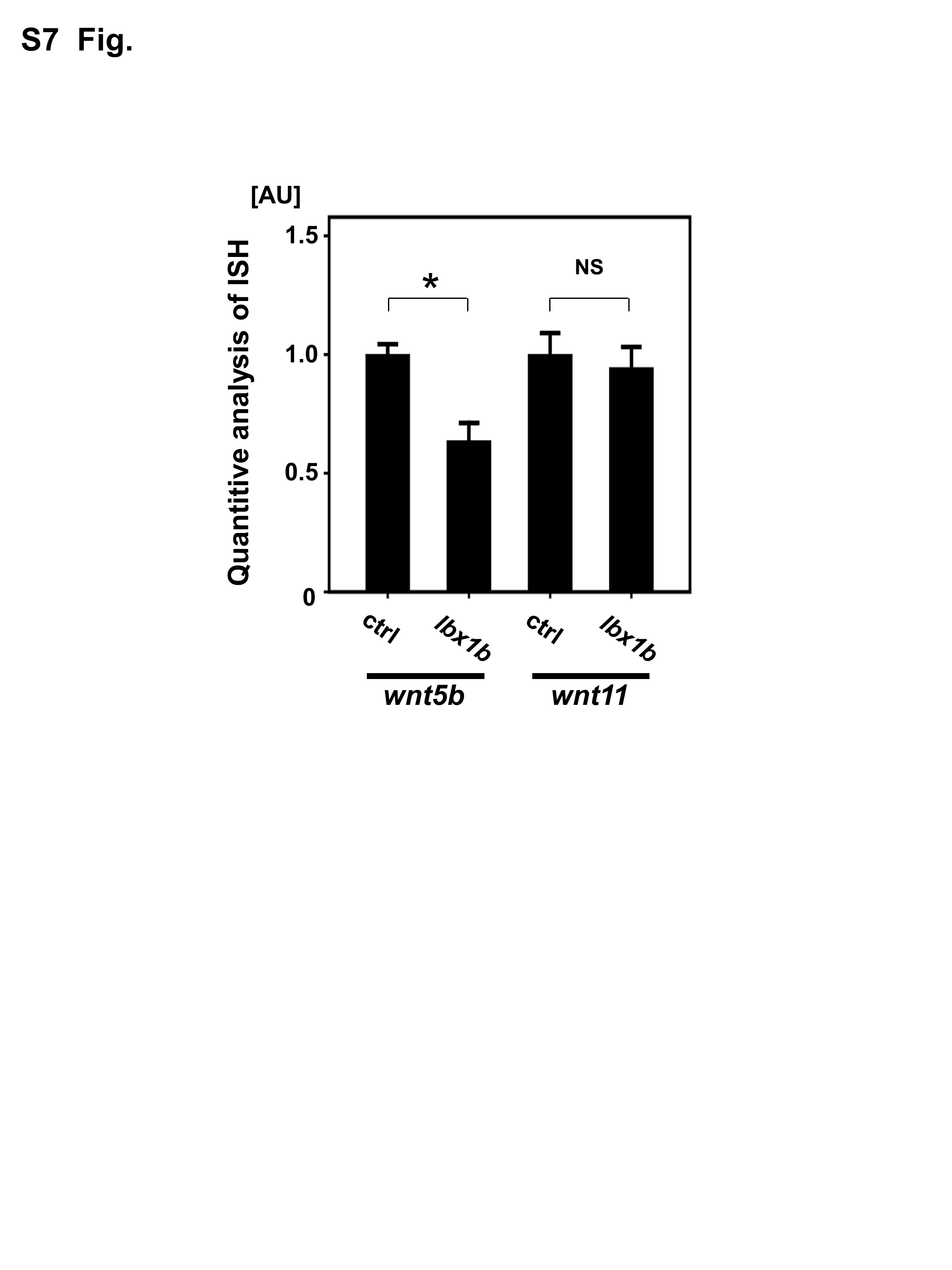

Supplement: S7 Fig — Quantitative analysis of the signal intensity of in situ hybridization for wnt5b or wnt11 by processing the images shown in Fig 6A. Significant differences (*p < 0.01) were observed in wnt5b. (TIF) [file pgen.1005802.s007.TIF]

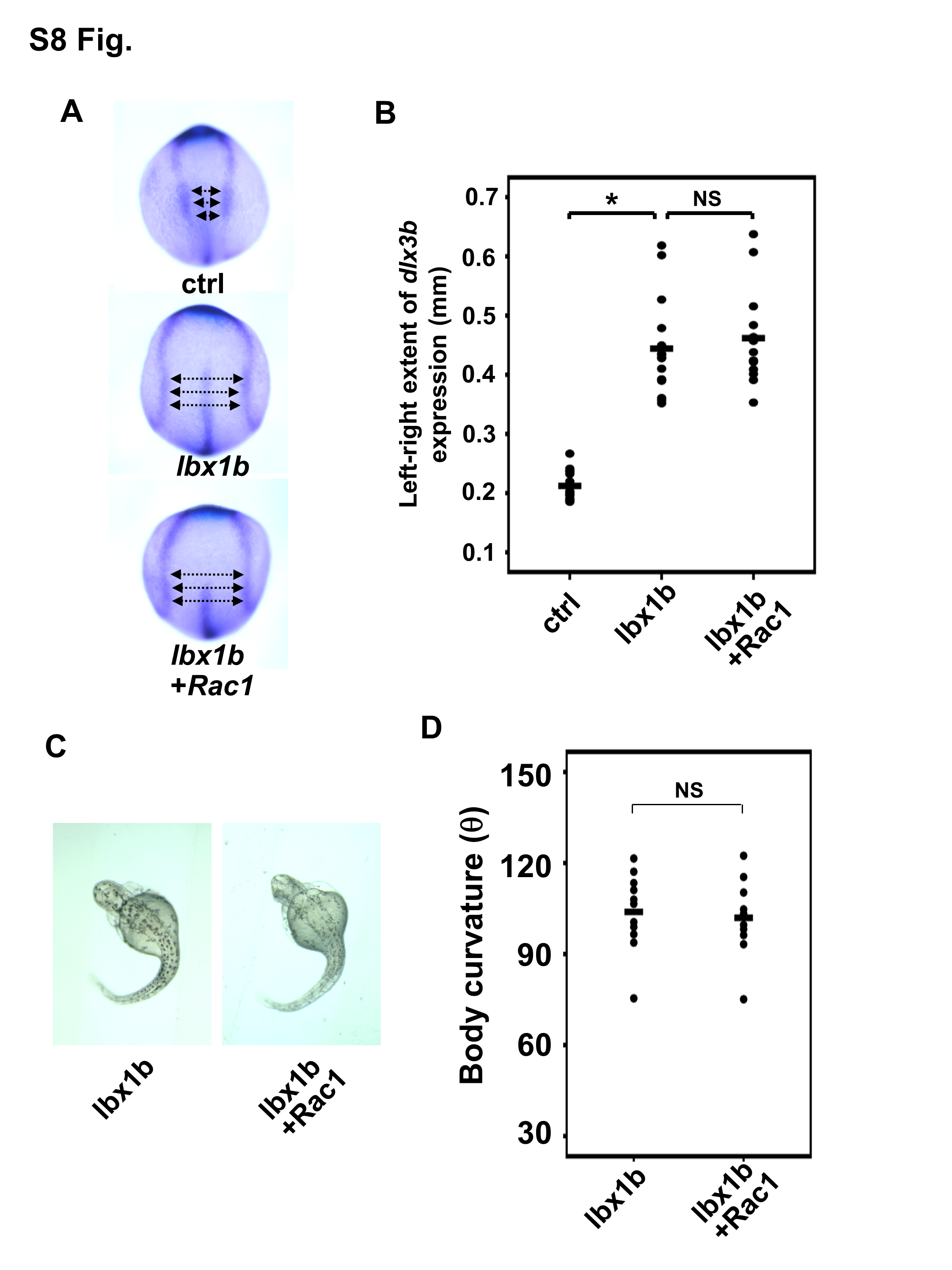

Supplement: S8 Fig — (A) Dorsal view of dlx3b/hgg1/ntl expression in the tail bud of embryos injected with buffer (ctrl), lbx1b mRNA (lbx1b), or lbx1b and human RAC1 mRNA (lbx1b+Rac1). (B) Quantitative analysis of convergent extension (CE) movement with embryos in (A) (ctrl, n = 15; lbx1b, n = 15; lbx1b+Rac1, n = 13). The extent of defective CE was evaluated by measuring the distance between the inner edges of bilateral dlx3b expression at 3 regions as indicated by the arrows in panel A. RAC1 mRNA injection failed to rescue defective CE. *p < 0.01, NS: not significant. (C) Dorsal view of 48 hpf Tg(hsp:Gal-VP; EGFP:UAS:lbx1b) embryos upon heat shock at 4 hpf with buffer (lbx1b) or RAC1 mRNA (lbx1b+Rac1) injection. (D) Quantitative analysis of body curvature with the embryos in (C) (lbx1b, n = 11; lbx1b+Rac1, n = 11). No significant change of the severity of body curvature was observed in RAC1 mRNA-injected embryos. NS: not significant. Severity of body curvature was quantified by the angle as described in Fig 3. F2 lines of driver and responder transgenic fish were used. The dosages for embryo injections were 50 pg/embryo for lbx1b mRNA and 40 pg/embryo for RAC1 mRNA. (TIF) [file pgen.1005802.s008.TIF]

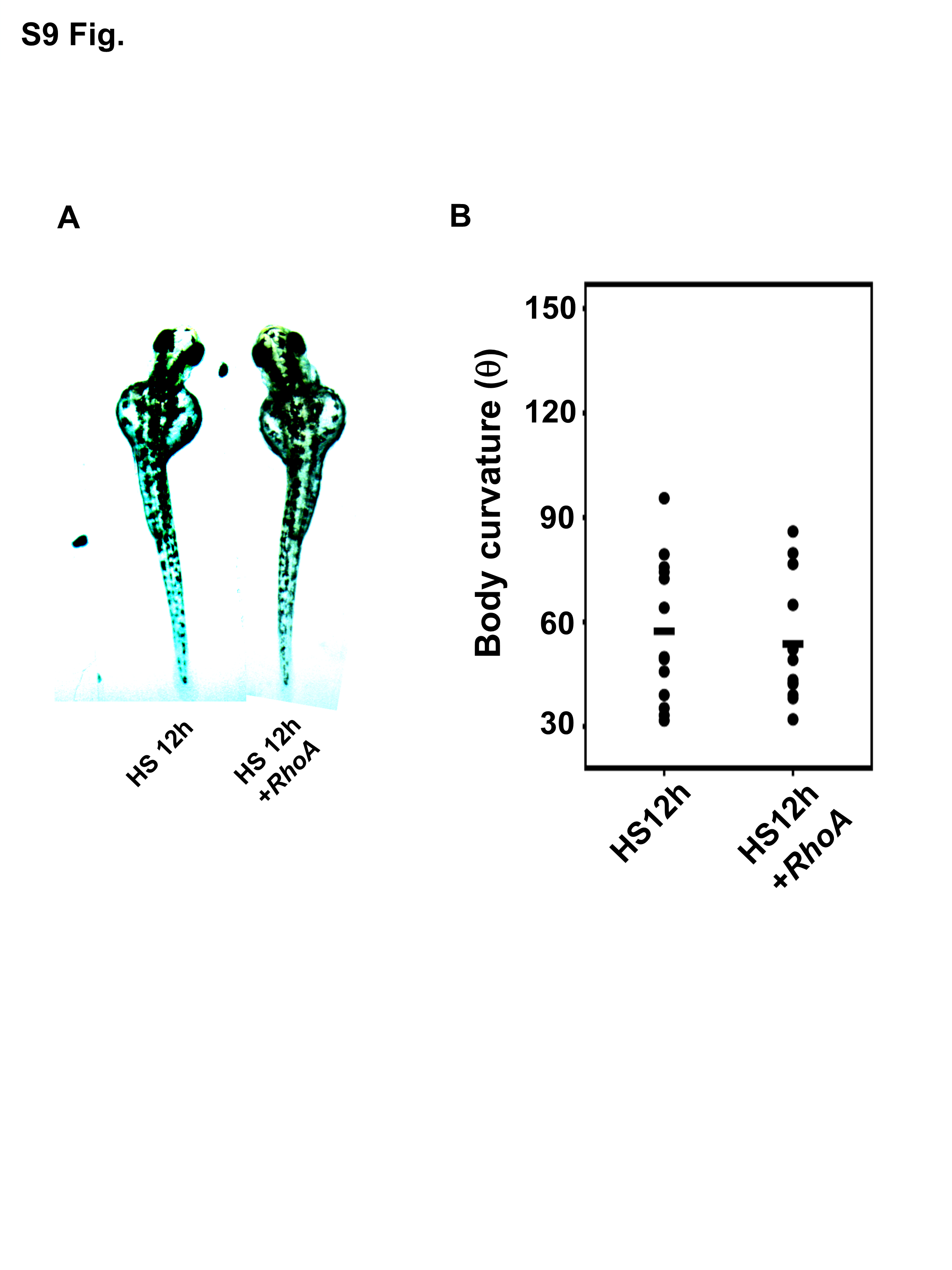

Supplement: S9 Fig — (A) Dorsal views of 48 hpf Tg(hsp:Gal-VP:EGFP:UAS:lbx1b) embryos upon heat shock (HS) at 12 hpf with buffer (HS12h) or RhoA mRNA (HS12h+RhoA) injection. (B) Quantitative analysis of body curvature in Tg(hsp:Gal-VP:EGFP:UAS:lbx1b) embryos upon HS at 12 hpf with buffer injection (lbx1b, n = 13) or RhoA mRNA injection (lbx1b+RhoA, n = 13). Significant change in axis development was not observed in RhoA-injected embryos with HS at 12 hpf. Severity of body curvature was quantified by the angle as shown in Fig 3E. F2 lines of driver and responder transgenic fish were used. (TIF) [file pgen.1005802.s009.TIF]

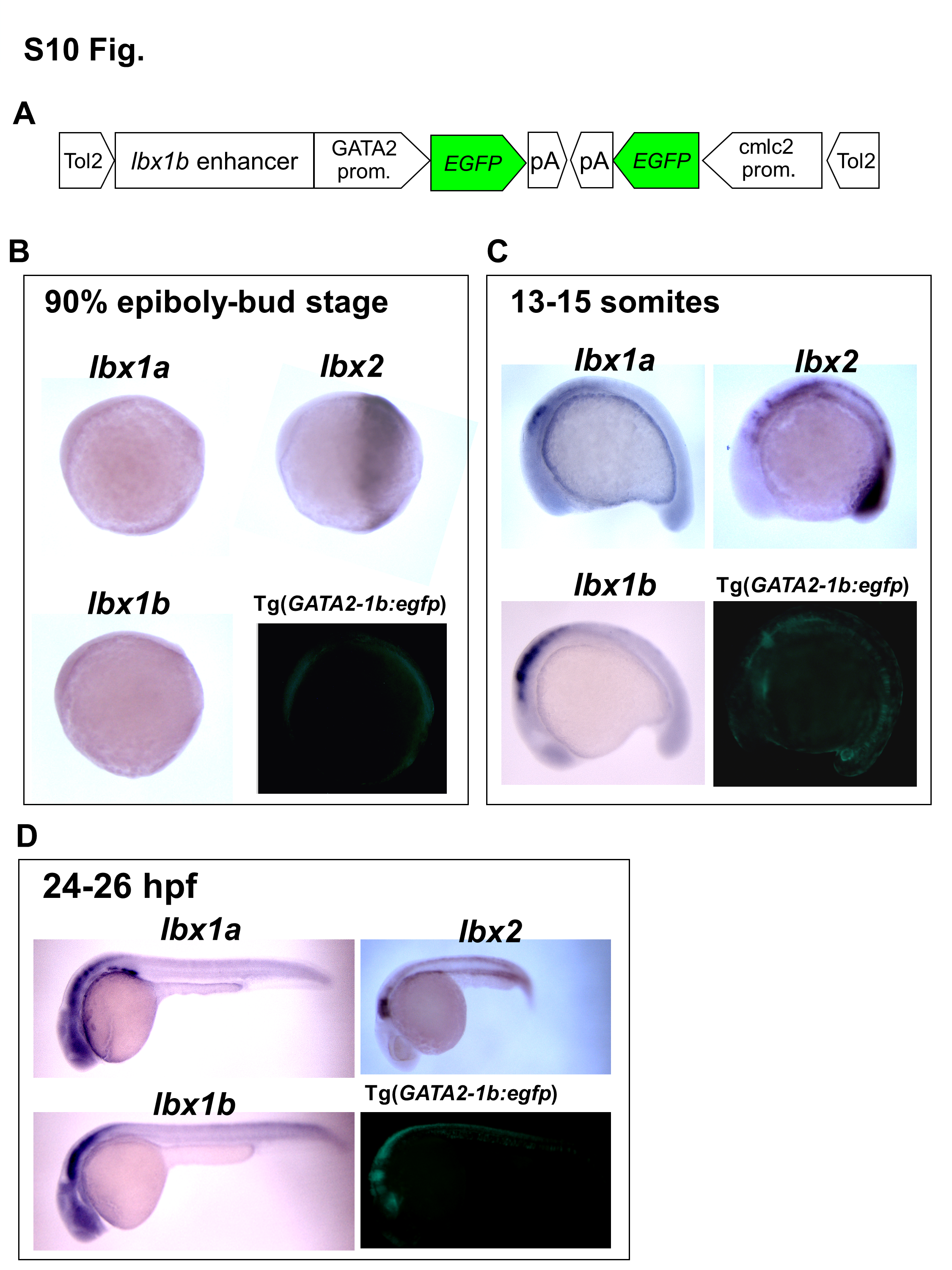

Supplement: S10 Fig — (A) Construction of the transgene. The GATA2 minimal promoter and an lbx1b enhancer cooperatively drive the expression of EGFP. The cardiac specific promoter cmlc2 drives EGFP expression in the heart as a transgenic marker. (B–D) Comparison of EGFP fluorescence with lbx1a, lbx1b, and lbx2 expression in lateral views at the 90% epiboly-bud stage (B), 13–15 somites stage (C), and 24–26 somites stage (D). (TIF) [file pgen.1005802.s010.TIF]

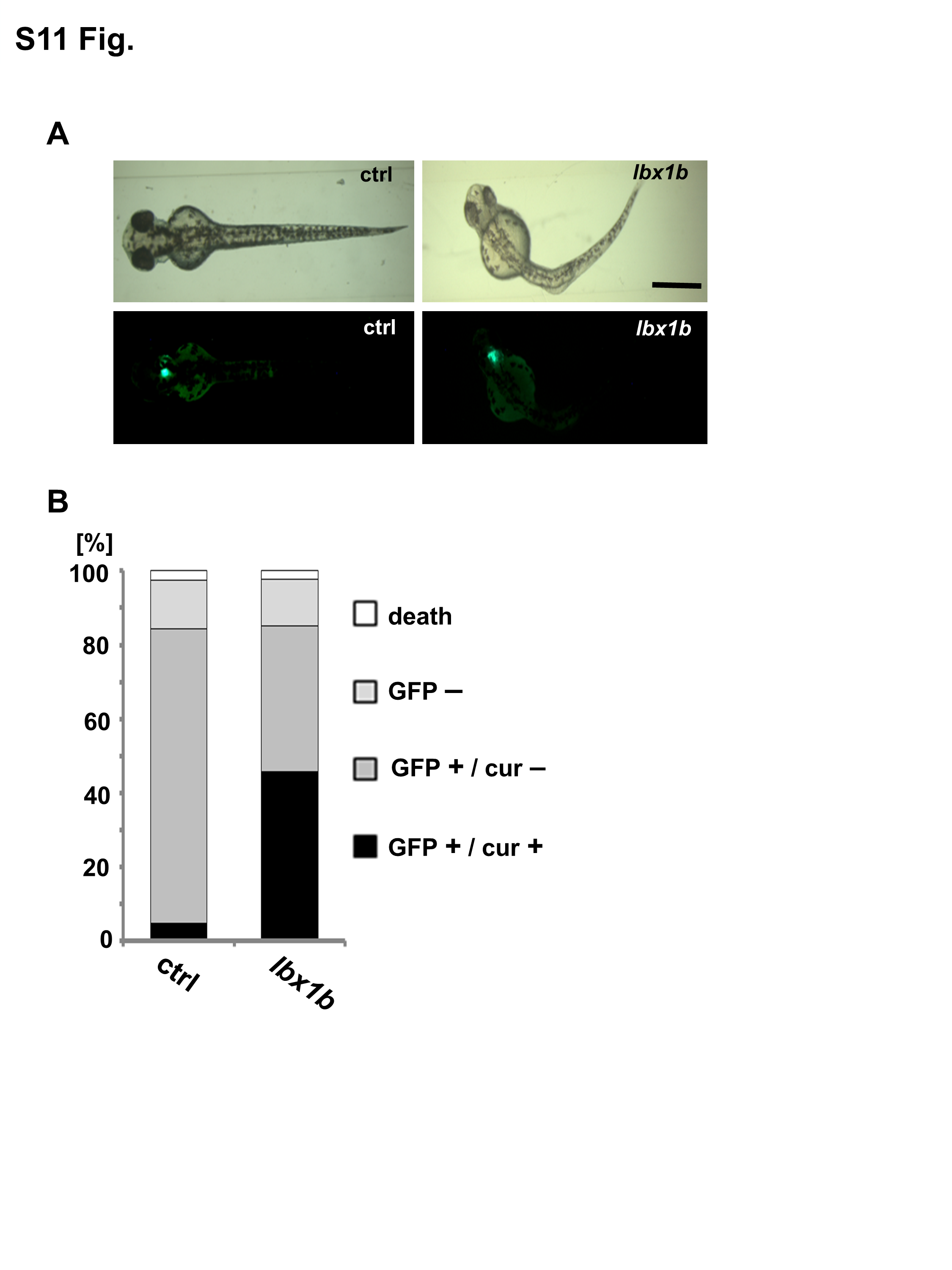

Supplement: S11 Fig — (A) Dorsal views of live embryos at 48 hpf. Body curvature was observed in zebrafish injected with GATA2-1b:lbx1b (lbx1b), but not in those injected with GATA2-1b:MCS (ctrl). The scale bar represents 500 μm. (B) Quantitative analysis of body curvature (cur) in 48 hpf embryos. The incidence of body curvature was significantly increased in lbx1b embryos (ctrl, 5%, n = 83; lbx1b, 46%, n = 94. p < 0.01). Scale bar in (A): 500 μm. (TIF) [file pgen.1005802.s011.TIF]

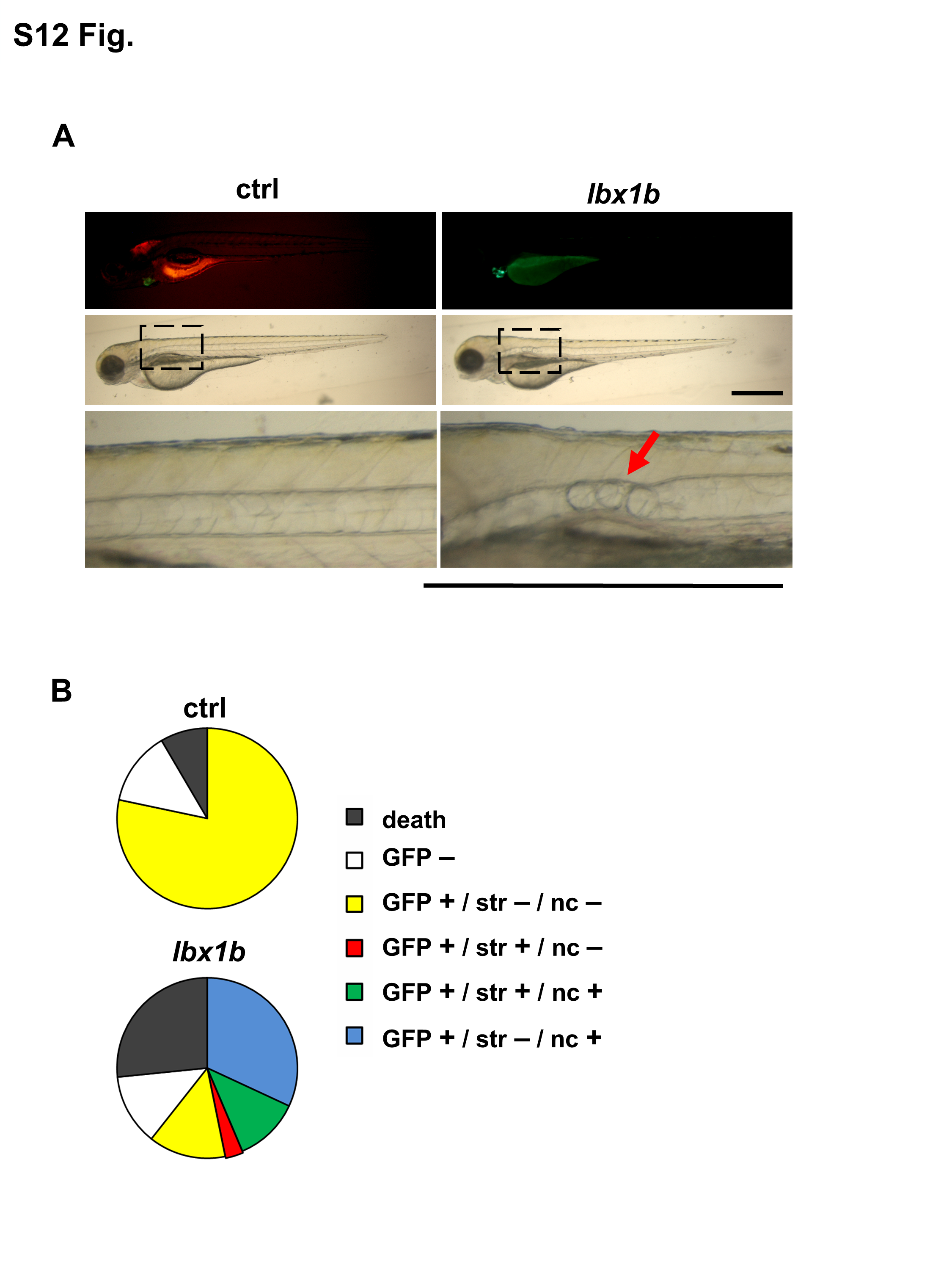

Supplement: S12 Fig — (A) Lateral views of embryos (48 hpf) injected with GATA2-1b:mCherry (ctrl) or GATA2-1b:lbx1b (lbx1b). Local notochord deformation (red arrow) was observed in lbx1b embryos. The lower panels show magnified views of the areas indicated by the dotted boxes in the corresponding middle panels. Scale bars represent 500 μm. (B) Quantitative analysis of the phenotypes of notochord deformation (nc+) and displaced dorsal melanophore stripe (str+) in 6 dpf zebrafish (ctrl, n = 224; lbx1b, n = 288). (TIF) [file pgen.1005802.s012.TIF]

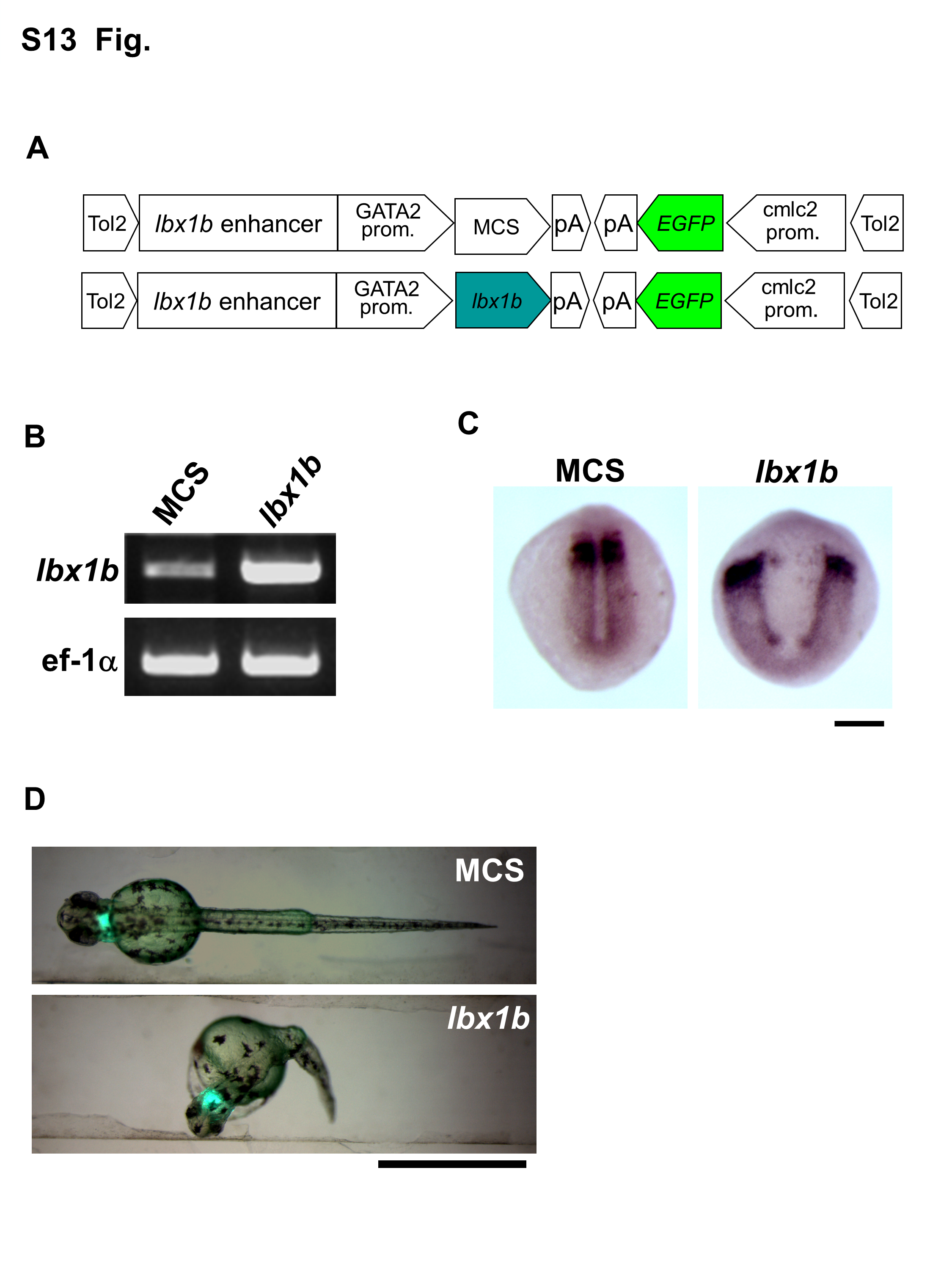

Supplement: S13 Fig — (A) The constructs used for transgenesis in zebrafish with the tol2 transposon system are shown. The cardiac specific promoter cmlc2 drives EGFP expression in the heart as a transgenic marker. (B) RT-PCR for 11 hpf embryos of Tg(GATA2-1b:MCS) F1 (MCS) and Tg(GATA2-1b:lbx1b) F1 (lbx1b) shows the elevated expression of lbx1b in lbx1b embryos. Ef-1α was used as a constitutive control. (C) Dorsal views of In situ hybridization for papc (paraxial mesoderm marker) at 11 hpf. Severe convergent defects were found in lbx1b embryos. The scale bar represents 200 μm. (D) Dorsal views of embryos at 48 hpf. Severe body curvature was observed in lbx1b embryos. The scale bar represents 1 mm. (TIF) [file pgen.1005802.s013.TIF]
