# Supplementary material for: Functional Investigation of a Non-coding Variant Associated with Adolescent Idiopathic Scoliosis in Zebrafish: Elevated Expression of the Ladybird Homeobox Gene Causes Body Axis Deformation
Source: PLoS Genet. 2016 Jan 28;12(1):e1005802. doi: 10.1371/journal.pgen.1005802 (PMC4731154; doi:10.1371/journal.pgen.1005802)
Supplement: S1 Table — (DOC) [file pgen.1005802.s014.doc]

**Table S1**

| **Name** | **Sequence (5’ to 3’)** |
| --- | --- |
| *lbx1a* forward | ATGACCTCCAAAGAAGACGCGAAAG |
| *lbx1a* reverse | TCAGTCATCTACATCAATTTCCTCGTCTTC |
| *lbx1b* forward | ATGACAACCATCAAAGTCGTCAAAATCTG |
| *lbx1b* reverse | TCAGACATCGACGTCTATTTCCACTTC |
| *lbx2* forward | GAATTCAtgacctccagctctaaagac |
| *lbx2* reverse | GAATTCttaatcgtccacctcgatttcc |
| *lbx1a*∆hd forward | gacctagaggagatgaaggcg |
| *lbx1a*∆hd reverse | ccgttttttaggggtattcctc |
| *lbx1a*∆eh forward | tctgtcaaacgaagttacac |
| *lbx1a*∆eh reverse | gagaggtttgtttgagttagc |
| *lbx1b*∆eh forward | actgtcaaacgatttcacag |
| *lbx1b*∆eh reverse | taagggcttgttggagctc |
| *lbx2*∆eh forward | tccgttaagaagtccgtcg |
| *lbx2*∆eh reverse | gagaggcttgttggagttg |
| *RhoA*_forward | gagcaatggctgccatccg |
| *RhoA _*reverse | caaggtttcacaagacaaggcacc |
| *Ef-1α_*qPCR_forward | CTGGAGGCCAGCTCAAACAT |
| *Ef-1α_*qPCR_reverse | ATCAAGAAGAGTAGTACCGCTAGCATTAC |
| *wnt5b_*qPCR_forward | cacgcacgcttatgaatctg |
| *wnt5b_*qPCR_reverse | aggttttcaagctgcacgag |
| *wnt5b_P1*_forward | ctcggcggccaagcttTCAGGCGAGGGGTTTTGTT |
| *wnt5b_P1_* reverse | ttggcatcttccatggCGCAACAGGTGTCAATTAA |
| *wnt5b_P2*_forward | ctcggcggccaagcttGTTTGTTTATTTATTTATT |
| *wnt5b_P2_* reverse | ttggcatcttccatggTTAGGAAATGATAGTAATC |
